# Panax-derived ginsenosides in diabetes-associated cognitive dysfunction: mechanistic evidence and translational barriers

**DOI:** 10.3389/fendo.2026.1965933

**Published:** 2026-09-08

**Authors:** Qi Yong, Xinrui Zhao, Yunxi Xu, Hua Bai, Hejiang Ye

**Affiliations:** 1 Affiliated Hospital of Chengdu University of Traditional Chinese Medicine, Chengdu, Sichuan, China; 2 Sichuan Key Laboratory of Traditional Chinese Medicine for Metabolic Diseases, Chengdu, Sichuan, China; 3 Clinical Medical College, Chengdu University of Traditional Chinese Medicine, Chengdu, Sichuan, China

**Keywords:** blood-brain barrier, brain insulin resistance, diabetes-associated cognitive dysfunction, ginsenosides, hippocampus, metabolic stress, neuroinflammation, target engagement

## Abstract

Diabetes-associated cognitive dysfunction (DACD) arises from interacting metabolic, vascular, inflammatory, and neuronal insults, but evidence for Panax-derived interventions is often overgeneralized across chemically non-equivalent materials. This critical narrative review evaluated whether defined Panax-derived saponins support a coherent mechanism of DACD and identified the evidence needed for translation. A structured multilingual search of eight bibliographic databases, supplemented by a targeted search of Cochrane CENTRAL, followed by deduplication and full-text reassessment, yielded 59 records: 16 A1 *in vivo* studies relevant to DACD (direct or near-direct), six neural-cell studies, 17 auxiliary Panax/barrier/formulation studies, and 20 contextual records. Purified monomers, the active metabolite compound K, saponin fractions, extracts, formulations, and combination interventions were appraised as non-interchangeable exposures. The most consistent signal was improved learning or memory accompanied by hippocampal or cortical tissue protection. Mechanistic findings converged on three connected but partly parallel processes: impaired brain insulin signaling and neuronal glucose handling; advanced glycation end product/methylglyoxal-associated oxidative and organelle stress; and glial-inflammasome amplification. Causal support was uneven, and brain exposure or central target engagement was rarely demonstrated. Most animal studies used young or adult animals of one sex and preventive or early-treatment designs, while several risk-of-bias domains were insufficiently reported. Panax-derived saponins should therefore be regarded as preclinical modulators of hippocampal metabolic-inflammatory-neuronal injury, not established DACD treatments. Translation requires defined interventions, parent/metabolite profiling, quantitative brain exposure, target-engagement assays, delayed-treatment and reversal designs, and clinically aligned biomarkers.

## 1 Introduction

(1–4) Type 2 diabetes mellitus (T2DM) affects the brain through metabolic, microvascular, inflammatory, and neurodegenerative processes that are not captured by peripheral glycemic indices alone. Diabetes is associated with modest cognitive decrements, mild cognitive impairment, and dementia, but these phenotypes differ in severity, age distribution, prognosis, and pathological composition. Experimental reports use the overlapping terms diabetes-associated cognitive dysfunction, diabetes-associated cognitive impairment, diabetes-associated cognitive decline, and diabetic encephalopathy. Here, diabetes-associated cognitive dysfunction (DACD) is used as an umbrella term; the other terms are retained when they identify a specific model or source literature.

(5–10) The central problem is how a systemic metabolic disorder becomes a disorder of learning and memory. Brain insulin resistance can impair neuronal glucose utilization and synaptic signaling; hyperglycemia increases advanced glycation end products (AGEs) and methylglyoxal (MGO); vascular injury weakens the blood-brain barrier (BBB); and oxidative, endoplasmic-reticulum (ER), and mitochondrial stress amplify glial and inflammasome responses. These processes converge particularly clearly in the hippocampus, where behavioral deficits in diabetic models are accompanied by neuronal loss, apoptosis, synaptic disturbance, gliosis, or Alzheimer disease (AD)-like changes. The hippocampus therefore provides the anatomical anchor that connects upstream metabolic stress to the cognitive phenotype.

Panax ginseng (11–16) C.A.Mey., Panax notoginseng (Burkill) F.H.Chen, and Panax quinquefolius L. are sources of dammarane-type saponins studied in metabolic and neurological disorders. The relevant interventions include purified ginsenosides Rg1, Rb1, Re, and Rg2; notoginsenoside R1 (NGR1); the intestinal metabolite compound K (CK); and chemically broader Panax notoginseng saponin (PNS), protopanaxatriol-type saponin (PTS), extract, and formula preparations. Broad reviews describe neuroprotective or antidiabetic activities of these materials, but they combine disease models, intervention layers, and evidence strengths that cannot be transferred directly to DACD.

The DACD-specific literature is considerably smaller than the separate bodies of literature on diabetes, cognition, or ginsenoside pharmacology considered separately. A clinical-efficacy claim would therefore exceed the evidence. The more defensible question is whether defined Panax-derived interventions connect cognitive behavior with central-tissue protection and whether their molecular findings form a biologically coherent injury network. This review tests that question through a sequence that follows the evidence itself: intervention identity and ethnopharmacological provenance, transparent evidence appraisal, the DACD disease framework, direct behavioral and tissue evidence, three connected mechanism chains, and finally the exposure, standardization, safety, and target-engagement barriers that determine whether preclinical effects can be translated.

## 2 Ethnopharmacological context and intervention identity

### 2.1 Botanical sources, medicinal parts, and traditional-use boundary

Accepted botanical names were verified against the World Flora Online Plant List on 14 July 2026 (17–19). The three Panax species are related but are not interchangeable medicines. Panax ginseng and P. notoginseng have established histories in East Asian materia medica, whereas P. quinquefolius is native to North America and entered Chinese medical practice through a later and distinct historical route. Current pharmacopoeial descriptions also separate the species, medicinal parts, processing, and indications (20).

Traditional descriptions of deficiency, wasting, thirst or fluid depletion, and spirit-calming or memory-related complaints explain why Panax materials became candidates for modern metabolic and neurological research. They do not show that DACD was a traditional diagnosis or that any Panax medicine had disease-specific cognitive efficacy. **Table 1** therefore separates documented source context from modern intervention claims. This boundary is central to the article’s logic: ethnopharmacology identifies what material is historically and botanically plausible, whereas experimental evidence determines what can be claimed for DACD.

**Table 1:** Panax sources, traditional context, and evidence boundaries relevant to this review.

Accepted botanical name and common/local name	Medicinal material	Traditional-use context and historical route	Relevant intervention layers	Evidence boundary
*Panax ginseng C.A.Mey. Asian/Korean ginseng; Ren Shen*	Root and rhizome; berry in selected modern studies	Established East Asian materia-medica use for deficiency and wasting, fluid depletion/thirst, and spirit-calming descriptions (15, 20)	Rg1, Rb1, Re and other dammarane saponins; extracts	Traditional use does not establish DACD efficacy, and extract evidence does not establish the effect of any purified ginsenoside.
*Panax notoginseng (Burkill) F.H.Chen Notoginseng; Sanqi*	Root and rhizome	Established Chinese use centered on hemostasis, stasis dispersal, and pain; raw and processed materials differ chemically (20, 24)	NGR1, Rg1, Rb1, PNS and PTS fractions	PNS/PTS evidence does not establish NGR1 or individual-monomer efficacy; NGR1 evidence does not establish fraction or whole-plant efficacy.
*Panax quinquefolius L. American ginseng; Xiyangshen*	Root; berry in selected modern studies	North American origin; later incorporation into Chinese practice for qi/yin deficiency and fluid depletion, with a historical route distinct from the Asian species (19, 20)	Re-rich and mixed saponin/extract preparations	Extract or herb-pair evidence does not establish purified Re or other monomer efficacy.

### 2.2 From plant material to administered intervention and active entity

(21–24) Botanical origin alone does not define the pharmacological exposure. Processing changes the relative abundance of common and rare saponins, and intestinal microorganisms can deglycosylate parent compounds into metabolites such as CK. Consequently, a root, processed product, extract, total-saponin fraction, purified monomer, and active metabolite represent different interventions. Their composition, bioavailability, and potential central exposure cannot be treated as equivalent.

**Figure 1:**
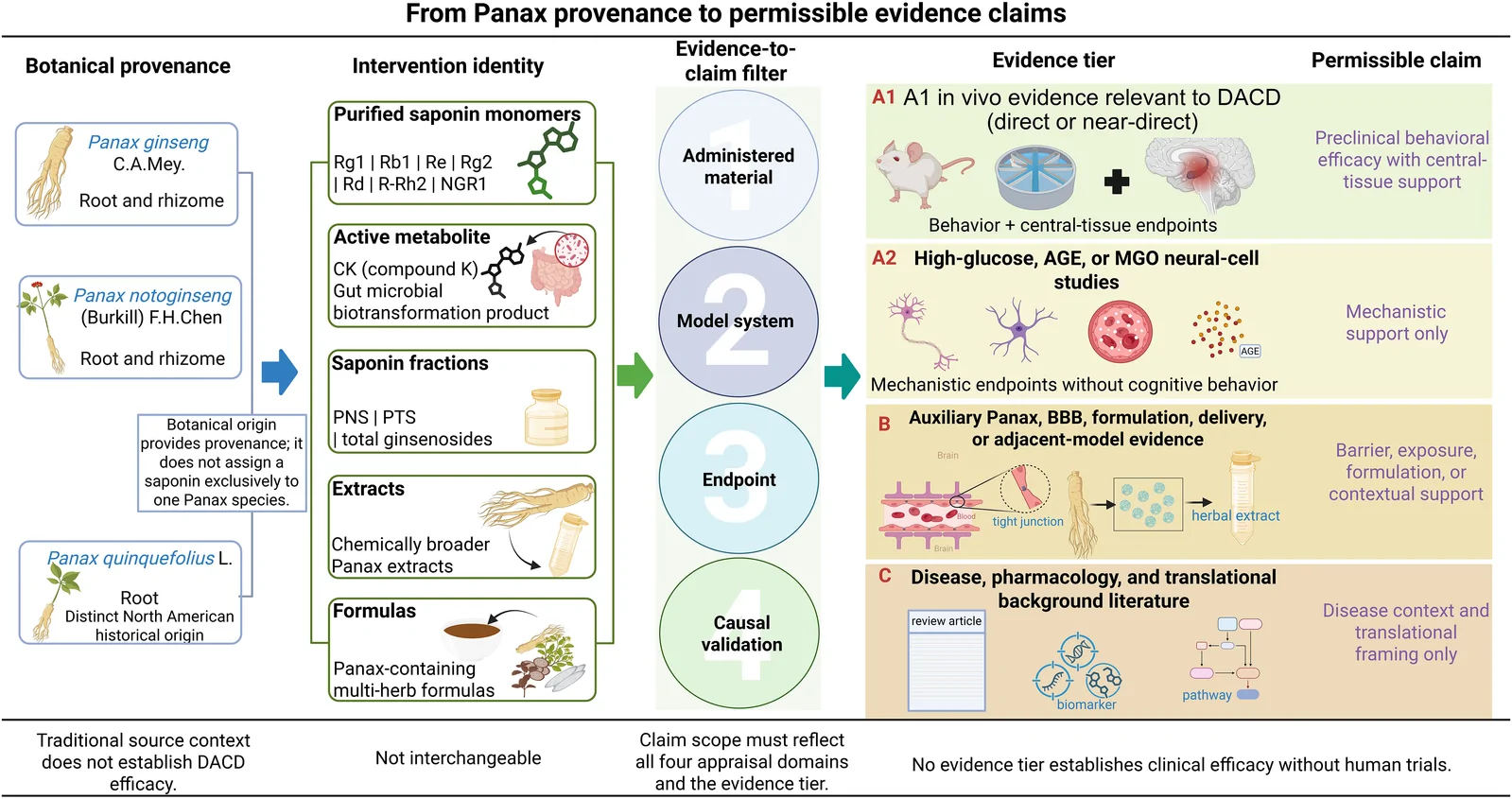
From Panax provenance to permissible evidence claims. Botanical provenance establishes source context but does not assign a saponin exclusively to one Panax species. Purified monomers, active metabolites, saponin fractions, extracts, and formulas are not interchangeable. Administered material, model system, endpoint, and causal validation are appraised before the evidence is assigned to A1, A2, B, or C. A1 supports preclinical behavioral claims only when central-tissue endpoints are present; A2 provides mechanistic support without cognitive efficacy; B and C provide auxiliary or contextual support. No evidence tier establishes clinical efficacy without human trials.

Mechanistic attribution therefore follows the administered material. Findings from CK cannot be assigned automatically to a parent ginsenoside; Rb1 combined with rosiglitazone does not establish Rb1 monotherapy; and effects of PNS, PTS, extracts, or formulas cannot prove the efficacy of a purified constituent. **Figure 1** integrates intervention identity with model system, endpoint, causal validation, and evidence tier. This evidence-to-claim filter links the ethnopharmacological starting point to the later mechanism sections by defining which chemical entity each experiment actually tests and how strongly the resulting claim can be stated.

## 3 Review strategy and evidence appraisal

### 3.1 Search, deduplication, and study selection

This critical narrative review used a structured multilingual search rather than a meta-analytic design. Eight principal databases were searched from inception to 3 July 2026 without language or publication-type restrictions: PubMed, Embase, Web of Science Core Collection, Scopus, China National Knowledge Infrastructure, Wanfang, VIP, and SinoMed. Cochrane CENTRAL was searched on the same date as a supplementary source and is not counted among the eight principal databases. Search concepts combined Panax, ginseng, notoginseng, ginsenoside, named saponins, or saponin fractions with diabetes-related terms and cognition, memory, hippocampal, neuronal, brain insulin resistance, neuroinflammation, or blood-brain barrier terms. Complete database-specific strategies are provided in **Supplementary Methods S1**. A targeted PubMed update was completed on 25 August 2026 (151 to 152 records); the single new record (PMID 42577123) was excluded at title/abstract screening and did not change the final 59-record evidence set.

The eight principal database searches yielded 1,710 records, and the Cochrane CENTRAL supplementary search yielded 2 records, totaling 1,712 records before deduplication. Duplicate detection used normalized DOI, PMID, title, author, and year fields, followed by record-level inspection of duplicate clusters; 660 records were removed, leaving 1,052 unique records, of which 1,028 had an abstract. QY and XZ screened titles and abstracts within a shared documented workflow. Potentially eligible or uncertain records were retained for focused eligibility reassessment, which involved title/abstract review and full-text checking where necessary. The same two authors jointly verified full-text eligibility. Differences in screening or eligibility judgments were resolved through discussion and consensus. The process was collaborative and sequential rather than two independent parallel screens; no claim of independent duplicate screening is made.

Eligible A1 studies used diabetes, insulin resistance, or a closely related metabolic model and reported cognitive behavior together with hippocampal or other central-tissue endpoints. A2 studies exposed neural cells to high glucose, AGE-related stress, or MGO and tested a DACD-relevant mechanism. Panax extracts, formulas, BBB/delivery studies, and adjacent brain models were retained only as auxiliary evidence. Reviews and disease-mechanism papers framed pathobiology, standards, or translation and were not used as proof of ginsenoside efficacy.

Evidence tiers were defined before synthesis. A1 comprised *in vivo* diabetes or insulin-resistance models that reported cognitive behavior together with hippocampal or other central-tissue endpoints. A2 comprised neural-cell studies using high glucose, AGE-related stress, or MGO to test a DACD-relevant mechanism; A2 evidence cannot establish cognitive efficacy. B comprised auxiliary Panax extracts, formulas, delivery or BBB studies, and adjacent neurological models. C comprised contextual disease-mechanism, pharmacokinetic, safety, standards, and review literature. A1 denotes direct or near-direct *in vivo* evidence relevant to DACD, not clinical evidence, and causal strength was judged separately within each tier. Adjacent or composite models remain within A1 only when cognitive behavior and central-tissue endpoints were reported together; they are not treated as standard DACD models.

**Figure 2:**
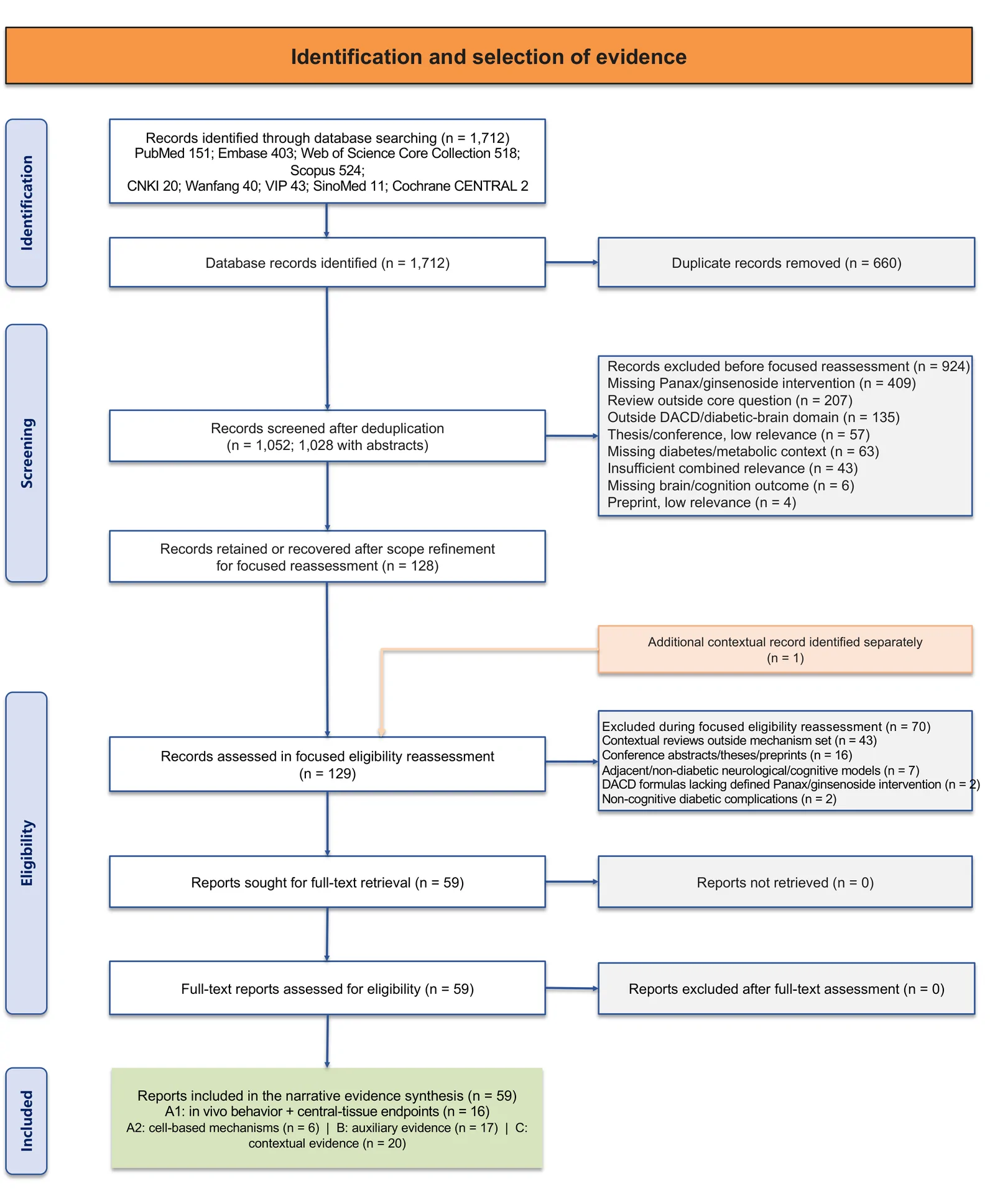
PRISMA-style flow diagram of evidence identification, deduplication, screening, focused eligibility reassessment, full-text confirmation, and evidence-tier inclusion for Panax-derived ginsenosides in diabetes-associated cognitive dysfunction. The eight principal database searches were completed on 3 July 2026 and Cochrane CENTRAL was searched as a supplementary source. A targeted PubMed update on 25 August 2026 identified one additional record (PMID 42577123), which was excluded during title/abstract screening and did not alter the final evidence set.

The documented selection process is summarized in **Figure 2**. Of the 1,052 unique records screened, 924 were excluded during initial screening and scope review. A total of 128 records were retained or recovered, and one contextual record identified separately was added, yielding 129 records for focused eligibility reassessment. Seventy records were excluded for the reasons shown in **Figure 2**. The remaining 59 reports were retrieved for full-text confirmation; none was excluded at that stage. The final evidence synthesis comprised 16 A1, six A2, 17 B, and 20 C records.

### 3.2 Data extraction, evidence classification, and quality appraisal

The final evidence inventory comprised 16 A1 *in vivo* studies, six A2 neural-cell studies, 17 auxiliary Panax/barrier/formulation studies, and 20 contextual records. For each A1/A2 study, QY and XZ jointly extracted intervention identity and purity, model, dose, route, duration, behavioral endpoint, central-tissue phenotype, mechanistic readout, causal perturbation, and principal limitation. Extraction was organized in a structured evidence matrix, and both authors cross-checked entries against the 59 source PDFs during drafting. Extraction or classification discrepancies were resolved through discussion and consensus. The records and their allowed functions are listed in **Supplementary Table 2**. Zhai et al., 2018 contains both A1 *in vivo* and A2 neural-cell components and is counted once in the 16-report A1 inventory; its cell findings are also appraised in the A2 series.

(25–27) Appraisal was adapted to evidence type and informed by established phytopharmacology reporting standards and the ConPhyMP guidance. Animal studies were examined for randomization, blinding, sample-size justification, diabetic phenotyping, cognitive testing, central-tissue endpoints, and pathway perturbation. Cell studies were examined for diabetes-like exposure, dose-response design, biological replication, compound definition, and inhibitor, knockdown, binding, or rescue experiments. Fractions, extracts, and formulas were additionally assessed for botanical authentication, processing, chemical profiling, and batch information. The four appraisal domains are reported in **Supplementary Table 3** as design/reporting, material definition, causal validation, and principal limitation; no unvalidated aggregate quality score was assigned.

(28) The 16 A1 animal studies also underwent a SYRCLE-informed domain-level risk-of-bias assessment covering sequence generation, baseline comparability, allocation concealment, random housing, blinding of personnel, random outcome assessment, blinded outcome assessment, incomplete outcome data, selective reporting, and other bias. Each domain was judged as low, high, or unclear risk; absence of reporting was classified as unclear rather than assumed to be inadequate. No aggregate score was calculated. Sex, age, sample-size justification, behavioral controls, and treatment timing were recorded as separate translational modifiers (**Supplementary Table 5**). Domain-level judgments are presented in **Supplementary Table 4**; sex, age, disease stage, sample-size justification, behavioral controls, treatment timing, and selective-reporting context are summarized in **Supplementary Table 5**.

In this review, target engagement means evidence that the administered compound or an identified metabolite physically or functionally engages the proposed target at an achieved exposure. A change in a downstream biomarker alone was classified as associative support, not target engagement. Preclinical behavioral improvement denotes better performance in an animal cognitive task; central-tissue protection denotes a reduction in tissue or molecular injury; and rescue is reserved for a formal rescue design.

Heterogeneity in species, diabetes induction, dose, treatment window, behavioral task, and molecular endpoint precluded a meaningful pooled effect estimate. The synthesis therefore asks first whether behavior and central tissue converge, then whether the proposed mechanisms are causally supported, and finally whether the administered entity could plausibly reach and engage the proposed central target.

## 4 Integrated pathobiological framework of DACD

### 4.1 From systemic metabolic disturbance to brain vulnerability

(2, 5, 6, 10) DACD is best understood as a multi-compartment process rather than as a single neuronal pathway. Systemic hyperglycemia, dyslipidemia, and insulin resistance alter cerebral microvessels, endothelial transport, glial metabolism, and neuronal fuel use. Brain insulin signaling supports synaptic plasticity and survival across neurons and non-neuronal cells; its impairment can reduce glucose utilization, disturb PI3K/Akt-GSK3β signaling and insulin-degrading enzyme (IDE), and increase susceptibility to Aβ- and tau-related stress. In parallel, diabetic microvascular injury and BBB dysfunction weaken neurovascular homeostasis and increase exposure of hippocampal tissue to inflammatory and metabolic signals.

### 4.2 From carbonyl stress to organelle failure

(8, 29–32) Persistent hyperglycemia also increases AGE formation and reactive dicarbonyl stress, particularly MGO. AGE/RAGE signaling and MGO can increase reactive oxygen species (ROS), impair protein homeostasis, and stress mitochondria and the ER. These insults are mutually reinforcing: mitochondrial dysfunction reduces energy reserve and increases ROS, whereas ER stress and defective selective autophagy impair removal of damaged proteins and organelles. Blood AGE/RAGE measurements are not yet consistent stand-alone predictors of cognitive decline, but the cellular carbonyl-stress pathway remains biologically credible.

### 4.3 Inflammatory amplification and the hippocampal phenotype

(7, 9, 33–37) Metabolic and organelle stress create the conditions for inflammatory amplification. ROS, calcium dyshomeostasis, TXNIP, NF-κB-dependent priming, danger signals, and BBB leakage can activate microglia, astrocytes, cytokine networks, and NLRP3- or NLRP1-related inflammasomes. Clinical inflammatory-marker studies support an association with cognitive impairment in T2DM but are heterogeneous and do not identify a single causal biomarker. The disease framework therefore consists of three connected, partly parallel processes: impaired brain insulin signaling, carbonyl/oxidative/organelle stress, and glial-inflammasome amplification. They converge on hippocampal neuronal apoptosis, synaptic dysfunction, AD-like molecular stress, and impaired memory rather than forming one obligatory linear pathway.

This convergence defines the test applied to the intervention literature. A change in blood glucose or an isolated cytokine is insufficient by itself; persuasive DACD evidence should connect treatment with cognition and central-tissue protection, then locate that effect within one or more of the three processes. The direct studies are evaluated on that basis before the individual mechanism chains are examined.

## 5 Direct preclinical evidence: cognitive behavior and central-tissue protection

The 16 A1 studies span high-fat diet (HFD)/streptozotocin (STZ)-induced type 2 diabetes mellitus (T2DM), db/db and KKay genetic diabetes, Goto-Kakizaki rats, STZ-associated insulin resistance, and a combined APP/PS1×db/db model. This diversity broadens biological coverage but also prevents the studies from representing a single DACD phenotype. The clearest common signal is not peripheral glucose lowering. It is the pairing of improved learning or memory with hippocampal or cortical protection after Rg1, Rb1, Re, NGR1, CK, total ginsenosides, PNS, or PTS treatment (38–49).

(38–49) The tissue endpoints provide different levels of closure. Rg1 studies paired water-maze or related behavioral outcomes with neuronal morphology, apoptosis, Aβ generation, calcium signaling, or inflammasome readouts. Re linked cognition to brain insulin resistance in HFD-fed mice and to hippocampal/cortical oxidative-inflammatory injury in STZ-diabetic rats. CK, NGR1, and Rb1 connected memory to ER stress/NLRP3, neuronal glucose uptake, or NMDAR/IDE-related signaling, respectively. Fraction-level PNS/PTS studies also reported hippocampal protection, apoptosis, or astrocyte-associated changes, but their chemical definition was substantially weaker.

(48–55) Causal strength varied more than the direction of the results. Fu et al. combined Rg1 with Trpc6 deletion, linking calcium dysregulation and TXNIP/NLRP3 signaling to behavior and mitochondrial apoptosis. NGR1 was supported by binding and reporter assays, a PPARγ inhibitor, neuronal GLUT4 translocation, and glucose-uptake measurements in a diabetic AD-like model. NGR1 pathway dependence was also tested with PI3K inhibition in high-glucose HT22 cells. At the cell level, Rg2-PDHE1α engagement was supported by surface plasmon resonance, cellular thermal shift assays, and gene silencing, whereas Rb1 studies used pathway inhibitors or rescue designs to interrogate PI3K/Akt, GSK3β, CHOP, and IDE. These A2 studies strengthen mechanism attribution but cannot establish cognitive efficacy.

(48, 56, 57) Several studies require narrower interpretation. The two KKay reports administered Rb1 with rosiglitazone, so the behavioral and insulin-signaling changes belong to combination treatment rather than to Rb1 alone. The APP/PS1×db/db model tests interaction between diabetic metabolism and established amyloid pathology and cannot represent all DACD. Early Ginseng radix and extract studies showed changes in dentate cell proliferation, hippocampal c-Fos, nitric-oxide synthase, AGE/RAGE signaling, or BBB injury, but they lacked either defined saponin attribution or a complete cognitive-behavioral endpoint (58–62). They document the evolution of the field, not monomer efficacy.

(63–70) Auxiliary studies extend disease context without changing this hierarchy. Ginseng berry or Panax quinquefolius-Acorus preparations, CSF metabolomics, and Panax-containing formulas support hippocampal metabolic, CREB/BDNF, Nrf2, or proteomic hypotheses. The Rg5 STZ-memory model and Rb1-berberine diabetic depression study identify adjacent neural effects but do not satisfy the DACD A1 definition. Specifically, the Rg5 study used intracerebroventricular STZ to induce a chemically driven memory-deficit/Alzheimer-like phenotype without establishing diabetes; it is therefore B-tier adjacent evidence rather than A1 DACD evidence.

Most A1 studies used male rodents, preventive or early-treatment designs, and relatively short windows. Blinding, sample-size justification, intervention purity, and direct brain exposure were inconsistently reported. The evidence consequently supports attenuation of DACD-like behavioral and tissue injury in preclinical models, not reversal of established human cognitive decline. **Table 2** makes the model, exposure, causal test, and principal limitation visible for each core study.

**Table 2:** Direct A1 *in vivo* and A2 neural-cell evidence supporting the mechanism framework.

Study	Intervention/material	Model/system, sex, and age	Treatment timing	Cognitive test and central phenotype	Mechanistic anchor	Mechanistic validation and strength	Principal limitation
Fu et al., 2026 (49)	Rg1, ≥98%	HFD/STZ T2DM; male mice, 8 weeks	Treatment after T2DM induction; delayed reversal not tested	MWM, Y-maze, hole-board/open-field; neuronal injury and mitochondrial apoptosis	TRPC6-CHREBP-TXNIP-NLRP3	Genetic pathway validation: Trpc6 knockout; direct Rg1-TRPC6 binding not shown	Male only; brain exposure and direct Rg1-TRPC6 binding not measured
Li et al., 2025 (48)	NGR1 monomer	APP/PS1×db/db; male mice, treatment from 4 months	Longitudinal early intervention; no delayed reversal cohort	MWM and object-recognition testing; neuronal glucose uptake and hippocampal injury	PPARγ-Akt-GLUT4	Direct-target plus functional perturbation: SPR/reporter assay and GW9662	Male offspring and a diabetic AD-like model; generalizability to DACD is limited
Su et al., 2024 (47)	Rg1 monomer	HFD/STZ T2DM; adult male SD rats	Treatment after model establishment; delayed reversal not tested	MWM; cortical/hippocampal oxidative injury and apoptosis	Oxidative stress and apoptosis	Descriptive, non-causal: dose response without pathway perturbation	Male only; randomization/blinding unclear; central and systemic effects not separated
Dong et al., 2023 (45)	Rg1, >98%	HFD/STZ T2DM; male C57BL/6 mice, age NR	Treatment after model establishment; delayed reversal not tested	MWM; neuronal lesions, calcium signaling, and Aβ-related readouts	PLC-CN-NFAT1	Associative, non-causal: multiple pathway and tissue readouts	Male only; blinding unclear; no inhibitor, knockdown, or binding assay
Huang et al., 2023 (46)	Rg1 monomer	HFD/STZ T2DM; male mice, 8 weeks	Treatment after model establishment; delayed reversal not tested	MWM; neuronal, senescence, and vascular morphology	MAPK-NLRP1	Associative, non-causal: neuronal/NLRP1 colocalization	Male only; blinding and sample-size justification unclear; pathway mainly associative
Li et al., 2020 (43)	CK, ≥98%	db/db; female mice, 7 weeks	Early treatment in young genetic diabetes	MWM and Y-maze; ER-stress/NLRP3-related brain endpoints	ER stress-NLRP3	Reporting-quality support, not causality: randomized groups and blinded behavior	Female only; no direct pathway perturbation or brain-exposure measurement
Yang et al., 2020 (44)	Rb1 monomer	STZ-associated insulin resistance; male mice, approximately 15 weeks	Treatment initiated with the STZ-associated model; prevention versus reversal unresolved	MWM and step-down task; hippocampal NMDAR/IDE-related signaling	Cdk5/p35-NMDAR-IDE	Associative, non-causal: convergent hippocampal signaling	Male only; high-dose STZ model; no target rescue or brain exposure
Tian et al., 2018 (42)	Total ginsenosides	Goto-Kakizaki diabetes; male rats, 13 weeks	Treatment in established genetic diabetes; aged disease not tested	MWM; brain oxidative/nitrosative and cytokine endpoints	Oxidative/nitrosative stress; cytokines	Descriptive, non-causal: behavior plus brain biochemistry	Male only; blinding/sample-size justification unclear; fraction composition unresolved
Zhai et al., 2018 (50)	NGR1 monomer	db/db; male mice, 7 weeks	Early treatment in young genetic diabetes	MWM; hippocampal oxidative stress and NLRP3-related injury	Akt/Nrf2-NLRP3	Pharmacological perturbation in A2 cells only: LY294002	Male only; causal perturbation was cell based; achieved brain exposure not measured
T. Zheng et al., 2018 (98)	PTS fraction	STZ diabetes; male SD rats, age NR	Treatment after diabetes confirmation; delayed reversal not tested	MWM including swimming-speed control; hippocampal apoptosis	Bax-associated apoptosis	Descriptive, non-causal: behavior plus histology	Male only; randomization/blinding unclear; dose not body-weight normalized
Kim et al., 2017 (41)	Re, >90%	HFD metabolic model; male mice, 4 weeks at baseline	Short-term metabolic intervention; established DACD reversal not tested	MWM, Y-maze, and passive avoidance; brain insulin-responsive signaling	Insulin-responsive signaling	Descriptive, non-causal: dose response	Male only; short metabolic model; direct central and peripheral effects not separated
Li et al., 2015 (40)	PTS fraction	STZ diabetes; male SD rats, 8 weeks	Treatment after diabetes confirmation; delayed reversal not tested	MWM; hippocampal astrocyte-associated endpoints	GFAP/GDNF	Descriptive, non-causal: behavior plus glial readouts	Male only, n=8/group; blinding/sample-size justification unclear; fraction characterization limited
Liao et al., 2014 (39)	PNS fraction	STZ diabetes; male Kunming mice, age NR	Treatment after diabetes confirmation; delayed reversal not tested	MWM; hippocampal morphology	Hippocampal neuroprotection	Descriptive, non-causal: behavior plus histopathology	Male only; blinding unclear; older PNS characterization does not meet current standards
Gao et al., 2014 (56)	Rb1 + rosiglitazone	KKay diabetes; male mice, 10 weeks	Treatment after hyperglycemic KKay phenotype; combination only	MWM; hippocampal PI3K/GSK3β/tau signaling	PI3K-GSK3β-tau	Combination dose stratification; no factorial interaction test	Male only; Rb1 cannot be separated from rosiglitazone without a monotherapy/factorial design
Mao et al., 2014 (57)	Rb1 + rosiglitazone	KKay diabetes; male mice, 10 weeks	Treatment after hyperglycemic KKay phenotype; combination only	MWM; hippocampal insulin/PI3K signaling	Insulin-PI3K signaling	Combination dose stratification; no factorial interaction test	Male only; Rb1 cannot be separated from rosiglitazone without a monotherapy/factorial design
Liu et al., 2012 (38)	Re, >90%	STZ diabetes; male SD rats, 10 weeks	Treatment immediately after diabetes confirmation; prevention/early treatment	MWM; hippocampal/cortical oxidative-inflammatory injury	TNF-α, MDA, GSH	Associative, non-causal: behavior plus two brain regions	Male only; blinding and causal perturbation unclear; systemic glucose lowering may contribute
Zhu et al., 2026 (55)	Rg2 monomer	High-glucose HT22 cells	*In vitro* exposure; no treatment-stage analogy	No cognitive test; neuronal viability and energy metabolism	PDHE1α	Direct-target A2 support: SPR, CETSA, and PDHE1α silencing	Cell-only evidence; 12.5-50 μM physiological relevance and brain exposure are unknown
Nan et al., 2019 (54)	Rb1 monomer	MGO-exposed SH-SY5Y cells	*In vitro* pretreatment; physiological exposure uncertain	No cognitive test; oxidative stress, mitochondrial dysfunction, and apoptosis	PI3K/Akt	Pharmacological pathway perturbation in cells: LY294002	Transformed cell line; no cognitive phenotype or achieved-exposure comparison
Gu et al., 2014 (52)	Rb1 monomer	High-glucose primary rat hippocampal neurons	*In vitro* exposure; no treatment-stage analogy	No cognitive test; Aβ secretion and insulin signaling	GSK3β-IDE	Pharmacological comparator support in cells: LiCl	Cell-only evidence; direct target engagement and physiological exposure are uncertain
Liu et al., 2014 (53)	Rb1 monomer	High-glucose primary hippocampal neurons	*In vitro* exposure; no treatment-stage analogy	No cognitive test; neurotoxicity and ER-stress readouts	GSK3β-CHOP	Pharmacological comparator support in cells: LiCl	Cell-only evidence; no genetic or direct-target validation
J.M. Chu et al., 2014 (77)	Rd and R-Rh2, ~98%	MGO-exposed primary astrocytes	*In vitro* exposure; physiological exposure uncertain	No cognitive test; astrocyte insulin signaling and apoptosis	IR/IRS-Akt; caspase/PARP	Descriptive, non-causal: dose response	Astrocyte-only evidence; 5-50 μM physiological relevance is uncertain
Liu et al., 2013 (51)	Rb1 monomer	High-glucose primary hippocampal neurons	*In vitro* exposure; no treatment-stage analogy	No cognitive test; apoptosis and mitochondrial injury	ER stress-CHOP	Associative, non-causal: multiple ER/mitochondrial readouts	Cell-only evidence; direct target engagement not shown

A1 occupies rows 1–16 and A2 rows 17-22. Detailed doses, routes, durations, and reporting appraisal are provided in **Supplementary Table 3**. MWM, Morris water maze; NR, not reported.

## 6 Brain insulin resistance and Alzheimer-like molecular overlap

(5, 6) The direct evidence places impaired neuronal metabolic competence at the beginning of the mechanistic sequence. Insulin signaling in the brain regulates glucose handling, synaptic plasticity, neurotransmission, and survival across neurons and glia. In DACD models, NGR1, Rb1, and Re connect this general biology to measurable cognitive and hippocampal outcomes. The shared process is partial restoration of glucose uptake, insulin-responsive signaling, IDE/Aβ handling, or NMDAR-related function rather than uniform activation of one receptor or kinase.

### 6.1 NGR1: PPARγ/Akt/GLUT4 and neuronal glucose uptake

(1, 48, 71) NGR1 provides the strongest target-oriented example. In APP/PS1×db/db mice, NGR1 enhanced PPARγ/Akt/GLUT4 signaling, neuronal GLUT4 membrane translocation, and glucose uptake, with improved cognitive performance. Direct binding and reporter assays supported PPARγ agonism, and GW9662 attenuated the effect, strengthening pathway attribution. The model is informative because diabetic metabolic stress and amyloid pathology coexist, but it also defines the boundary: the result demonstrates a diabetic AD-like context, not equivalence between DACD and AD. Human evidence likewise indicates that vascular injury, insulin resistance, AGEs, and neurodegeneration contribute beyond amyloid and tau burden alone.

### 6.2 Rb1: GSK3β-IDE and Cdk5/NMDAR-related signaling

(44, 52, 56, 57, 72, 73) Rb1 links insulin signaling to IDE, Aβ, tau, and synaptic receptors across complementary models. In high-glucose hippocampal neurons, Rb1 altered GSK3β signaling, increased IDE, and reduced Aβ secretion. In STZ-associated cognitive impairment, oral Rb1 improved memory and regulated Cdk5/p35-NMDAR-IDE signaling. The KKay studies reported PI3K/GSK3β/tau and insulin-signaling changes only when Rb1 was combined with rosiglitazone. Alignment across the cell, monotherapy, and combination studies supports the pathway family, but only the monotherapy study supports Rb1-specific behavioral attribution. General PI3K/Akt and Rb1 reviews provide biological context rather than additional DACD efficacy evidence.

### 6.3 Re and the direct-versus-systemic attribution problem

(38, 41, 74, 75) Re complements this evidence at the level of whole-animal metabolism. In HFD-fed mice, Re improved cognitive performance and brain insulin-responsive signaling after four weeks of treatment. In STZ-diabetic rats, Re improved water-maze performance while reducing hippocampal and cortical TNF-α and lipid-peroxidation indices. Because both studies also changed systemic metabolic measures, they do not fully separate a direct central action from secondary benefit after peripheral improvement. Re-focused and antidiabetic Rb1 reviews reinforce this unresolved pharmacological distinction.

(76) Recent studies showed that Rg1 or an Rg1-containing formula can modify hypothalamic energy homeostasis, autophagy, inflammation, and central insulin resistance in glucolipid disorders. These findings support a central metabolic-inflammatory connection but are not hippocampal DACD evidence. The mechanistic sequence must therefore move to the next question: how persistent metabolic signaling failure is converted into cellular injury within memory circuits.

## 7 Carbonyl, oxidative, and organelle stress in hippocampal vulnerability

### 7.1 AGE/MGO stress and loss of neural metabolic support

(29, 30, 54, 77) Hyperglycemia converts signaling dysfunction into injury through overlapping carbonyl and redox stress. MGO is a reactive AGE precursor that can impair insulin signaling, damage proteins, and promote ROS and apoptosis. In SH-SY5Y cells, Rb1 pretreatment reduced MGO-induced oxidative stress, mitochondrial dysfunction, and apoptosis through a PI3K/Akt-dependent mechanism; LY294002 weakened the protection. In primary astrocytes, the specific ginsenosides Rd and R-Rh2 improved insulin signaling and reduced caspase/PARP-associated apoptosis after MGO exposure. This result is specific to Rd and R-Rh2, and the astrocyte system supports a mechanism of impaired neural support rather than cognitive efficacy.

(38, 47, 50, 61, 68) Animal studies connect the same stress domain to cognition. Re reduced TNF-α and MDA in the hippocampus and temporal cortex of diabetic rats, Rg1 reduced oxidative injury and neuronal apoptosis in HFD/STZ rats, and NGR1 coupled Akt/Nrf2 activation to lower oxidative stress and NLRP3 activity in db/db mice and high-glucose HT22 cells. Nrf2 is therefore more informative as an adaptive stress-response link between redox control and inflammatory restraint than as a generic antioxidant label. Ginseng extract acting through AGE/RAGE-NF-κB and a Panax quinquefolius-Acorus preparation acting through Nrf2-Keap1 remain auxiliary support because their active entities are unresolved.

### 7.2 ER stress, mitochondrial dysfunction, and energy failure

(43, 51, 53, 78, 79) High-glucose primary hippocampal-neuron studies place ER stress downstream of metabolic overload. Rb1 at 1 μM reduced high-glucose neurotoxicity, GSK3β-associated CHOP induction, mitochondrial dysfunction, and apoptosis. CK extended the ER-stress link to behavior: 12 weeks of oral CK improved memory in db/db mice while reducing ER stress and NLRP3-related signaling. Because CK is an intestinal metabolite, this evidence belongs to CK and cannot be reassigned to a nominal parent compound.

(55) Rg2 provides the most direct energy-metabolic target evidence. In high-glucose-injured HT22 cells, Rg2 bound and stabilized PDHE1α, improved pyruvate-dehydrogenase activity, ATP and acetyl-CoA production, and redox balance, and lost part of its effect after PDHE1α silencing. This moves the field beyond nonspecific ROS suppression toward a testable mechanism linking glycolytic carbon entry, mitochondrial energy production, and neuronal survival. It remains A2 evidence until brain exposure and cognitive benefit are demonstrated in a DACD animal model.

### 7.3 Organelle stress as the transition to inflammation

(8, 31, 33) ER dysfunction, mitochondrial ROS, impaired autophagy, calcium imbalance, and AGE/RAGE signaling can all increase TXNIP or NF-κB-dependent inflammasome readiness. Carbonyl and organelle stress therefore explain how persistent metabolic failure lowers hippocampal resilience; they do not end the injury chain. Once glial and inflammasome responses become self-amplifying, the dominant question shifts from cellular stress to propagation of inflammatory damage.

## 8 Inflammasome and glial amplification of neuronal injury

### 8.1 Rg1: TRPC6-NLRP3 and MAPK-NLRP1 evidence

(49) The most complete behavior-to-mechanism example in the current corpus is the Rg1 TRPC6-TXNIP-NLRP3 study. HFD/STZ-induced T2DM produced aberrant TRPC6 activation, calcium overload, CHREBP nuclear accumulation, TXNIP upregulation, NLRP3 activation, mitochondrial apoptosis, neuronal injury, and cognitive dysfunction. Rg1 reduced these changes, while Trpc6 deletion provided an upstream genetic perturbation. Calcium dyshomeostasis and TXNIP therefore precede inflammasome activation in this model, whereas apoptosis and neuronal dysfunction connect the molecular cascade to behavior.

### 8.2 NGR1, CK, and Re: convergent but non-equivalent inflammatory evidence

(38, 42, 43, 46, 50, 80) The available studies do not support one universal inflammasome target. A separate Rg1 study linked improved memory and neuronal/vascular morphology to lower MAPK-NLRP1 signaling, although the pathway remained mainly associative. NGR1 linked Akt/Nrf2 activation to NLRP3 restraint, with PI3K inhibition reducing the cell-level protection. CK connected ER-stress reduction to lower NLRP3 activity in db/db mice, while Re and total ginsenosides produced broader cytokine and oxidative/nitrosative changes without isolating a specific inflammasome mechanism. A recent medicinal-chemistry synthesis similarly identifies inflammasome/pyroptosis regulation as a cross-complication hypothesis, not as proof that every ginsenoside shares the same target.

### 8.3 Glial localization and unresolved cell specificity

(40, 77) Cellular localization remains less mature than molecular pathway mapping. PTS altered astrocyte-associated GFAP/GDNF readouts in diabetic rats, and Rd/R-Rh2 protected MGO-exposed primary astrocytes (34, 81–84). A Wumei Pill study identified an astrocyte-derived CXCL1/CXCR2 route in diabetic encephalopathy, and recent work emphasizes microglial metabolic reprogramming as a determinant of inflammatory output. These sources define experiments that the ginsenoside field still needs; they do not establish that a purified saponin acts through the same cell-specific route. HMGB1 and CX3CL1 literature likewise supports plausible danger-signal and neuron-glia communication modules without proving ginsenoside-specific target engagement in DACD.

**Figure 3:**
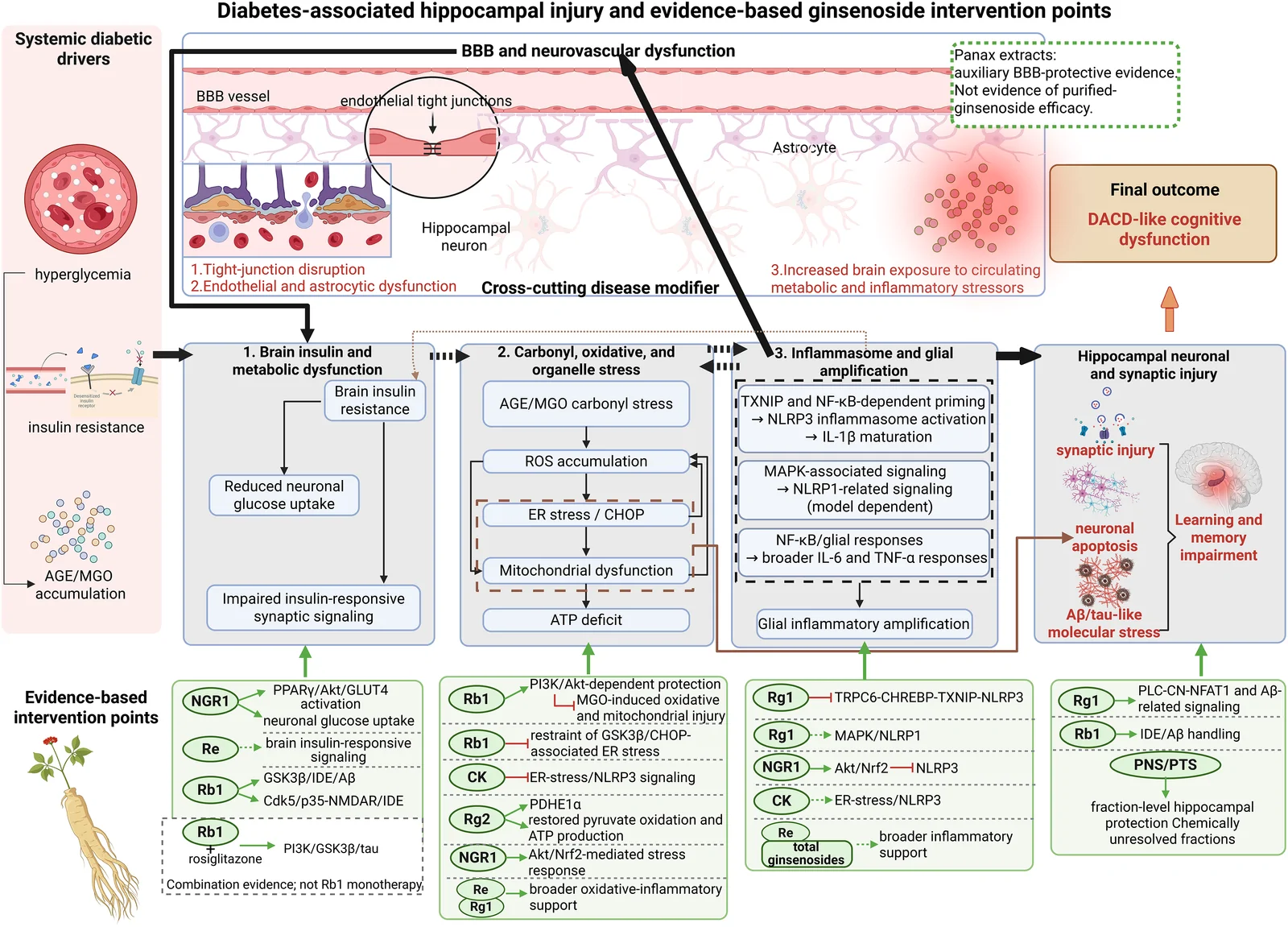
Diabetes-associated hippocampal injury and evidence-based ginsenoside intervention points. Systemic hyperglycemia, insulin resistance, and AGE/MGO accumulation influence three connected and partly parallel processes: brain insulin and metabolic dysfunction; carbonyl, oxidative, and organelle stress; and inflammasome/glial amplification. BBB and neurovascular dysfunction act as a cross-cutting disease modifier. These processes converge on hippocampal neuronal and synaptic injury, model-dependent Aβ/tau-like molecular stress, learning and memory impairment, and DACD-like cognitive dysfunction. Black solid arrows indicate within-study or strongly established disease-mechanism links; black dashed arrows indicate cross-study integration or incompletely validated links. Green solid arrows denote activation or restoration of protective processes supported by direct or pharmacological perturbation evidence; green dashed arrows denote associative, auxiliary, or broader support. T-shaped connectors denote inhibition of pathological processes. PNS/PTS effects are shown as chemically unresolved fraction-level evidence. Not every connection was validated within the same model.

The current evidence therefore locates Rg1, NGR1, CK, Re, and saponin fractions at partially distinct points in an amplification process: metabolic or organelle stress primes inflammatory signaling; glial and inflammasome responses propagate injury; and neuronal apoptosis or synaptic failure produces the behavioral phenotype. Cell-resolved, spatial, and lineage-specific perturbation studies are required to distinguish direct neuronal actions from microglial, astrocytic, endothelial, or barrier-mediated effects. **Figure 3** integrates the three mechanism chains, and **Table 3** states the intervention-specific boundaries.

**Table 3:** Compound-pathway relationships, evidence strength, and attribution boundaries.

Intervention	Best-supported action	Evidence domain	Attribution boundary	Evidence strength
Rg1	Restrains TRPC6-TXNIP-NLRP3 and MAPK-NLRP1; modulates PLC-CN-NFAT1	Inflammasome amplification, calcium-related injury, Aβ-related stress	Pathways are model specific; TRPC6 dependence is not direct binding	Genetic pathway support for TRPC6; other Rg1 pathways mainly associative
Rb1	Supports PI3K/Akt/IDE and restrains GSK3β-CHOP-related injury	Insulin/IDE signaling, MGO injury, ER stress	Two *in vivo* reports used rosiglitazone combination treatment	A2 pharmacological perturbation plus associative A1 evidence
Re	Improves brain insulin-responsive signaling and oxidative-inflammatory indices	Brain insulin resistance and redox/inflammatory stress	Direct central action is not separated from systemic metabolic benefit	Associative *in vivo* support
NGR1	Activates PPARγ/Akt/GLUT4 and Akt/Nrf2; restrains NLRP3	Neuronal glucose uptake and oxidative-inflammasome coupling	Strong target-oriented evidence remains preclinical	Direct-target/pharmacological support for PPARγ; cell-level PI3K perturbation
CK	Reduces ER stress and NLRP3 signaling	ER stress/inflammasome and cognition	Metabolite evidence cannot be assigned to a parent ginsenoside	Associative *in vivo* support
Rg2	Binds/stabilizes PDHE1α and improves energy metabolism	Mitochondrial substrate use and neuronal viability	A2 cell evidence; *in vivo* DACD validation is absent	Direct-target plus gene-silencing A2 support
Rd/R-Rh2	Improve insulin signaling and reduce apoptosis after MGO	Astrocyte metabolic support	A2 astrocyte evidence only	Descriptive A2 support
PNS/PTS	Reduce hippocampal injury, apoptosis, or glial abnormalities	Fraction-level behavioral and tissue protection	Insufficient chemical characterization in older studies	Descriptive *in vivo* fraction-level support
Extracts/formulas	Support BBB, AGE/RAGE, metabolic, and cell-interaction hypotheses	Auxiliary disease and delivery context	Cannot establish purified-ginsenoside efficacy	Auxiliary/contextual support

## 9 BBB injury, brain exposure, and central target engagement

### 9.1 The BBB as a disease compartment

(2, 10, 62, 85, 86) The BBB belongs inside the DACD injury network rather than outside it. Hyperglycemia, insulin resistance, and microvascular dysfunction can injure endothelial cells, alter tight-junction proteins and transport systems, disrupt pericyte and astrocyte-endfoot support, and facilitate inflammatory signaling into hippocampal tissue. Ginseng extracts reduced BBB disruption and hippocampal neuronal apoptosis in STZ-diabetic rats, supporting barrier involvement but not the efficacy of a purified ginsenoside. BBB injury can therefore amplify neuronal vulnerability while simultaneously changing the route by which an administered compound reaches the brain.

### 9.2 The BBB as a compound-specific exposure constraint

(21, 22, 43, 78) Oral exposure cannot be inferred from nominal dose. Parent ginsenosides differ in intestinal transformation, absorption, protein binding, clearance, and tissue distribution. Processing and the gut microbiota can change the administered saponin profile before systemic exposure occurs. CK is the clearest example: it is an active metabolite with its own pharmacology, and its db/db cognitive evidence must remain assigned to CK.

(13, 14, 23, 75, 87–89) The exposure problem is also compound specific. NGR1 reviews describe low oral bioavailability and limited CNS penetration, motivating intranasal, nanoparticle, or permeability-enhancing strategies. Rg1 has broad neurological activity, but its delivery and central pharmacokinetics remain formulation dependent. In diabetic rats with cerebral infarction, an Rg1 nanoparticle increased BBB penetration and improved cerebral outcomes; this result demonstrates the contribution of formulation and exposure, not conventional oral Rg1 efficacy in DACD. Reviews of Re, Rb1, and general ginsenoside pharmaceutics provide useful compound-specific background, but they do not replace measurements in the model under study.

Diabetes can also alter the pharmacokinetic context itself. Disease-related changes in gastrointestinal motility, intestinal and hepatic drug-metabolizing enzymes, microbial deglycosylation, plasma protein binding, renal clearance, BBB integrity, and transporter expression may change parent-to-metabolite ratios and central exposure. The direction and magnitude are compound-, model-, sex-, and disease-stage-dependent; pharmacokinetic findings from healthy animals therefore cannot be transferred directly to DACD models (22, 23).

### 9.3 From detected exposure to central target engagement

(90–92) Most A1 DACD studies measured behavior and brain signaling without quantifying parent compound or metabolite concentrations in plasma, CSF, and brain. Consequently, a central molecular change may reflect direct brain action, secondary improvement in systemic metabolism, or both. Future experiments should identify the circulating and central chemical entities first, establish an exposure-response relationship second, and test target engagement at achievable concentrations third. General delivery reviews reached a similar conclusion but necessarily combine disorders and preparations.

(48, 49, 55) The current corpus already shows what stronger validation looks like. NGR1-PPARγ binding and pharmacological antagonism connect a defined compound to a functional metabolic target in a brain-relevant model. Rg2-PDHE1α surface plasmon resonance, thermal stability, and silencing establish direct cell-level engagement, although *in vivo* exposure is unknown. Trpc6 deletion strengthens the Rg1 pathway chain but does not by itself demonstrate direct Rg1 binding to TRPC6. Distinguishing pathway dependence from physical drug-target engagement prevents a plausible mechanism from being presented as a demonstrated central pharmacological interaction. **Table 4** separates DACD pharmacodynamic findings from measured systemic exposure, brain exposure, and central target engagement for each intervention layer.

**Table 4:** Evidence for systemic exposure, brain exposure, and central target engagement.

Intervention/material	DACD pharmacodynamic evidence	Systemic PK evidence	Brain/CSF measurement in DACD	Central target engagement	Permissible interpretation
Rg1	A1 behavior plus central-tissue signaling	General/formulation literature only	Not quantitatively measured in the A1 studies	Trpc6 genetic dependence; no direct Rg1-TRPC6 binding	Direct central action remains unresolved
Rb1	A1 monotherapy and combination studies; A2 neural-cell studies	General compound-level literature	Not quantitatively measured in the DACD studies	Cell-level pathway perturbation; no achieved-exposure central binding	Separate monotherapy from rosiglitazone combinations
Re	A1 cognitive and brain signaling/oxidative-inflammatory outcomes	General compound-level literature	Not quantitatively measured in the A1 studies	Not demonstrated	Direct central action cannot be separated from systemic benefit
NGR1	A1 behavior, neuronal glucose uptake, and oxidative-inflammasome outcomes	Low oral bioavailability/limited CNS context from adjacent literature	Not linked quantitatively to the DACD effects	PPARγ binding/reporter/antagonist support; achieved brain exposure not linked	Strong target-oriented preclinical evidence with an exposure gap
CK	A1 behavior and ER-stress/NLRP3-related outcomes	Active-metabolite literature	Not quantitatively measured in the A1 study	No direct central binding test	Evidence belongs to CK, not an assumed parent ginsenoside
Rg2	A2 neural-cell evidence only	No DACD *in vivo* exposure evidence	Not measured	PDHE1α SPR/CETSA and silencing in cells	Direct cell-level engagement requires *in vivo* DACD validation
PNS/PTS/total ginsenosides	A1 fraction-level behavior plus tissue outcomes	Constituent-specific PK unresolved	No constituent-resolved measurement	Not demonstrated	Effects remain assigned to the administered fraction
Extracts/formulations/formulas	Auxiliary BBB, delivery, metabolic, or adjacent-model evidence	Preparation-specific	Formulation-dependent and not transferable	Generally not demonstrated for a defined saponin	Cannot establish purified-ginsenoside efficacy

A1, *in vivo* evidence relevant to DACD (direct or near-direct); A2, neural-cell mechanistic evidence; CSF, cerebrospinal fluid; PK, pharmacokinetics. ‘Not measured’ refers to the DACD study set and does not imply absence from all disease or healthy-animal literature.

## 10 Discussion

### 10.1 Material standardization, safety, and limits of evidence transfer

(26, 27) The transition from ethnopharmacological source to reproducible intervention depends on material definition. Purified compounds require identity, stereochemical assignment where relevant, purity, impurity profile, and batch information. Fractions and extracts additionally require the accepted botanical name, medicinal part, voucher or authentication, processing and extraction conditions, chromatographic fingerprint, and quantified marker compounds. Formulas require all ingredients and their quality controls. Several older PNS/PTS studies did not meet these current standards, so their behavioral results cannot be mapped onto a single constituent.

(18, 23, 78, 87, 88) Safety is equally material specific. Reviews of P. ginseng and P. quinquefolius describe a generally favorable short-term record while also noting product variability, adverse effects, and potential interactions; those observations cannot establish the long-term safety of repeated central exposure to a defined monomer or metabolite in older people with diabetes. NGR1, CK, and modern formulation reviews emphasize that altered exposure and delivery can change both efficacy and safety, so a delivery system cannot inherit the safety assumptions of an oral botanical product. The A1 literature provides little long-term toxicology, drug-interaction, or aged-comorbidity evidence.

Specific safety questions include possible ginseng-warfarin interactions and altered coagulation, estrogen-like effects, rare hepatotoxicity reports, and interactions with glucose-lowering or central nervous system drugs. These signals are inconsistent and often arise from heterogeneous products or case reports; they should not be presented as uniform toxicity of every purified ginsenoside (18). Dose-response may also be nonlinear or biphasic. A concentration that is protective in one cell or exposure window cannot be assumed to remain beneficial at higher or prolonged exposure, particularly when free brain concentrations and active metabolites are unknown.

(12, 16, 22, 72, 91–95) Evidence transfer is a separate source of inflation. Reviews of ginsenosides in AD, stroke, general neurodegeneration, or PNS neurological applications summarize useful pharmacology, but their disease models and exposures are not substitutes for DACD. Likewise, historical extract effects, formula studies, and adjacent neurological models cannot be converted into monomer efficacy. Head-to-head comparison under the same DACD model is needed before activity is transferred among parent compound, metabolite, fraction, extract, and formula. A recent middle-aged-rat study comparing aerobic conditioning, ginseng supplementation, and their combination illustrates why parallel improvements do not by themselves demonstrate pharmacological synergy; because it was neither a diabetes model nor a chemically defined monomer study, it remains adjacent evidence for factorial and interaction-based design rather than DACD efficacy.

(96) Clinical evidence remains the final boundary. A Cochrane review did not establish reliable cognitive benefit from ginseng products, and no controlled clinical study currently demonstrates efficacy of a chemically defined ginsenoside for DACD. Thus, material standardization, exposure, and safety are not secondary reporting issues; they determine whether the preclinical mechanism can be reproduced and tested in humans.

### 10.2 Evidence limitations and sources of heterogeneity

The evidence base is constrained by both study design and review process. Most A1 studies used male rodents; one used females, and few directly tested sex-dependent effects. Young or adult animals and preventive or early interventions predominated, so the literature does not establish reversal of persistent DACD in aged, multimorbid animals. HFD/STZ, db/db, KKay, Goto-Kakizaki, STZ-associated insulin-resistance, and APP/PS1×db/db models represent different metabolic and neuropathological contexts and cannot be ranked across studies without matched head-to-head experiments. Reporting of allocation concealment, random housing, sample-size justification, and blinded outcome assessment was frequently unclear. QY and XZ both participated in title/abstract screening, full-text verification, and data extraction, but these tasks were performed within one collaborative documented workflow rather than as two independent parallel reviews. Consensus discussion, full-text rechecking, and evidence-matrix cross-checking reduced but could not eliminate selection and classification bias, and they do not provide the same protection against reviewer dependence as independent duplicate screening.

### 10.3 Translational priorities and decisive experiments

The evidence gaps define an ordered translational program. The administered material must first be authenticated and chemically specified. Parent and metabolite concentrations must then be measured in plasma, CSF, and brain to identify the active exposure. Target engagement should be tested at those concentrations by binding, CETSA, inhibitor, knockdown, knockout, or rescue designs. Only after identity, exposure, and engagement are established can compounds be compared fairly in standardized DACD models. Reversing this sequence risks optimizing a behavioral signal without knowing which entity or target produced it.

(37, 95) Model standardization should include at least one insulin-resistant T2DM model, a prespecified memory task with locomotor and sensory controls, hippocampal or cortical tissue endpoints, both sexes where feasible, and delayed-treatment designs that distinguish prevention from reversal. Cell-resolved analysis should determine whether neuronal, microglial, astrocytic, endothelial, or pericyte responses are primary. Multi-omics can nominate pathways, but causal perturbation is needed before an omics association becomes a mechanism. Middle-aged-rat comparisons of aerobic conditioning, ginseng supplementation, and their combination illustrate how age and factorial design can separate monotherapy, combination, and interaction effects.

(32, 35, 71, 84, 97) A human bridge requires biomarkers that reflect the same process measured in the animal model. Candidate domains include insulin signaling, AGE/RAGE burden, inflammasome-related inflammation, neuronal injury, BBB integrity, cerebral microvascular imaging, and hippocampal structure or function. None is sufficiently specific to function alone; prespecified multimodal panels and clinically defined cognitive phenotypes are preferable. Experience with GLP-1 receptor agonists illustrates the broader standard: pathway plausibility becomes clinically meaningful only when exposure, population, outcome, and biomarker are aligned.

Clinically aligned studies should prespecify memory, executive function, and processing-speed outcomes rather than use an undifferentiated cognition label. Candidate bridge measures include plasma and, where feasible, CSF exposure; neuronal injury markers such as neurofilament light and GFAP, interpreted as non-specific; hippocampal structural or functional imaging; cerebral perfusion and small-vessel or BBB-permeability measures; and a multimodal insulin/AGE-RAGE/inflammasome panel. PK, target engagement, biomarkers, and cognition should be measured within the same protocol so that a biological signal can be linked to an achieved exposure and a clinically defined phenotype.

**Figure 4:**
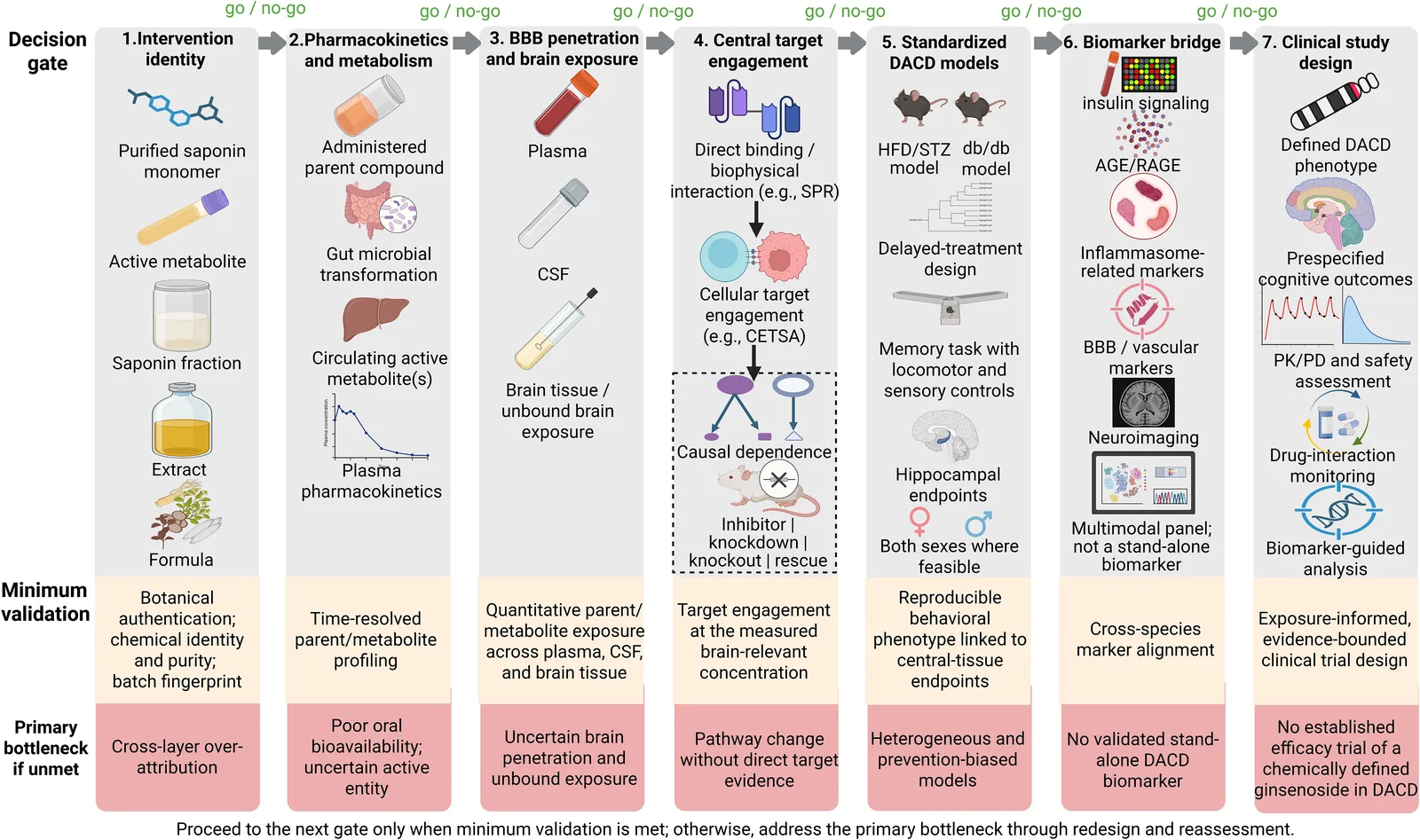
Translational decision pathway for Panax-derived saponin interventions. The sequential gates address intervention identity, parent/metabolite pharmacokinetics, BBB penetration and brain exposure, central target engagement, standardized DACD models, cross-species biomarker alignment, and clinically bounded study design. Progression to the next gate requires its minimum validation criterion to be met; otherwise, the primary bottleneck should be addressed through redesign and reassessment. Attribution control is a cross-cutting requirement: factorial or interaction designs should separate monomer, metabolite, fraction, and formulation effects, as illustrated by a middle-aged-rat aerobic-conditioning-plus-ginseng comparison (95). The sequence represents a go/no-go research pathway rather than a validated biological pathway.

**Table 5** and **Figure 4** convert these requirements into a staged decision pathway. A negative result at any early gate should redirect compound selection or formulation before a clinical study is attempted.

**Table 5:** Translational decision gates and experimental priorities.

Decision gate	Core question	Minimum recommended readouts	Reason for the sequence
Material identity	What botanical material and chemical entity were administered?	WFO name, medicinal part, authentication, purity/fingerprint, quantified markers, batch	Prevents substitution and cross-layer attribution
Active exposure	Which parent compound or metabolite is present systemically and centrally?	Time-resolved plasma, CSF, and brain quantification	Identifies the entity that can plausibly cause the effect
Central target engagement	Does that entity engage the proposed target at achieved exposure?	SPR/CETSA or equivalent binding plus inhibitor, knockdown, knockout, or rescue	Distinguishes target engagement from correlated pathway change
DACD model validation	Is efficacy reproducible across clinically relevant disease contexts?	Standardized T2DM models, delayed treatment, memory task with controls, central tissue endpoints, both sexes	Separates prevention from reversal and reduces model drift
Attribution control	Is activity due to monomer, metabolite, fraction, or formulation?	Head-to-head testing in the same model with matched endpoints	(95) Prevents transfer of efficacy among non-equivalent interventions. Factorial or interaction designs should separate monotherapy, combination, and interaction effects; the middle-aged-rat aerobic-conditioning-plus-ginseng comparison is a concrete example.
Biomarker bridge	Can the same biological process be measured in animals and humans?	Multimodal insulin, AGE/RAGE, inflammation, BBB, neuronal injury, and imaging panel	Links mechanism to a clinically defined phenotype
Safety and human validation	Is long-term exposure tolerable and clinically meaningful?	Interaction and chronic-toxicity assessment; prespecified cognition and biomarker outcomes	No defined-ginsenoside DACD efficacy has been established clinically

## 11 Conclusions

The direct preclinical literature supports a coherent but bounded conclusion: Panax-derived ginsenosides and saponin fractions can modulate DACD-like cognitive and central-tissue injury. The strongest evidence links impaired brain insulin signaling, AGE/MGO-associated oxidative and organelle stress, and inflammasome/glial amplification to hippocampal neuronal and synaptic damage. Rg1, Rb1, Re, NGR1, CK, Rg2, PNS, and PTS act at partially distinct points, and evidence cannot be transferred among monomers, metabolites, fractions, extracts, or combination regimens. This synthesis is nevertheless dominated by one-sex, young-to-adult, preventive or early-treatment animal experiments and should not be interpreted as evidence for reversal of established DACD in older patients.

The principal uncertainty is no longer whether these compounds alter preclinical readouts; it is whether a chemically defined active entity reaches the brain, engages the proposed target at a realistic exposure, remains safe during long-term use, and improves a clinically defined DACD phenotype. Until those requirements are met, ginsenosides should be described as preclinical modulators of hippocampal metabolic-inflammatory-neuronal injury rather than as established treatments.

## 12 Compounds and intervention layers reviewed

Purified compounds: ginsenosides Rg1, Rb1, Re, Rg2, Rg5, Rd, and R-Rh2, and notoginsenoside R1. Active metabolite: compound K. Saponin fractions: total ginsenosides, Panax notoginseng saponins, and protopanaxatriol-type saponins. Auxiliary interventions: chemically broader Panax extracts, delivery systems, and Panax-containing formulas.
