# Supplementary material for: Panax-derived ginsenosides in diabetes-associated cognitive dysfunction: mechanistic evidence and translational barriers
Source: Front Endocrinol (Lausanne). 2026 Sep 8;17:1965933. doi: 10.3389/fendo.2026.1965933 (PMC13595345; doi:10.3389/fendo.2026.1965933)
Supplement: Supplementary file 1 [file SupplementaryFile1.docx]

# Supplementary Material

## Supplementary Methods S1. Search strategy and database-specific queries

Search framework and verified database-specific strings. The review used one integrated concept search per database. The strings below reproduce the search records used for the historical baseline on 3 July 2026. No language or publication-type restrictions were applied; each database was searched from inception through the actual search date. The eight principal databases were PubMed, Embase, Web of Science Core Collection, Scopus, CNKI, Wanfang, VIP, and SinoMed; Cochrane CENTRAL was searched as a supplementary source.

Search status. The historical baseline comprises 1,712 records before deduplication: 1,710 from the eight principal databases plus 2 from Cochrane CENTRAL as a supplementary source. A targeted PubMed update on 25 August 2026 returned 152 records; the single new record (PMID 42577123) was excluded at title/abstract screening and does not change the final 59-record evidence set. The rows below report the verified historical hit counts as the final counts for the eight principal databases. The two historical Cochrane CENTRAL records were excluded at title/abstract screening and are not counted among the 59 retained reports.

| Database | Historical date | Historical hits | Search status | Evidence-base impact |
| --- | --- | --- | --- | --- |
| PubMed | 3 July 2026, 10:48 | 151 | Targeted update completed 25 Aug 2026 (NCBI E-utilities cross-check; 151 to 152) | New PMID 42577123 excluded; 59-record set unchanged |
| Embase | 3 July 2026, 10:56 | 403 | Historical baseline final (3 July 2026) | Historical hit retained as final count |
| Web of Science Core Collection | 3 July 2026, 11:42 | 518 | Historical baseline final (3 July 2026) | Historical hit retained as final count |
| Scopus | 3 July 2026, 11:48 | 524 | Historical baseline final (3 July 2026) | Historical hit retained as final count |
| CNKI | 3 July 2026, 12:00 | 20 | Historical baseline final (3 July 2026) | Historical hit retained as final count |
| Wanfang | 3 July 2026, 12:02 | 40 | Historical baseline final (3 July 2026) | Historical hit retained as final count |
| VIP | 3 July 2026, 12:03 | 43 | Historical baseline final (3 July 2026) | Historical hit retained as final count |
| SinoMed | 3 July 2026, 12:06 | 11 | Historical baseline final (3 July 2026) | Historical hit retained as final count |
| Cochrane CENTRAL | 3 July 2026, 11:57 | 2 | Supplementary source; historical status confirmed | Historical 2 records excluded at title/abstract screening |

### S1.1. PubMed: verified single search string

```
(
  "Ginsenosides"[Mesh]
  OR "Panax"[Mesh]
  OR ginsenoside*[tiab]
  OR notoginsenoside*[tiab]
  OR panaxoside*[tiab]
  OR "ginseng saponin*"[tiab]
  OR "Panax saponin*"[tiab]
  OR "Panax ginseng saponin*"[tiab]
  OR "Panax notoginseng saponin*"[tiab]
  OR "notoginseng saponin*"[tiab]
  OR "total saponins of Panax notoginseng"[tiab]
  OR PNS[tiab]
  OR "ginsenoside Rg1"[tiab]
  OR "ginsenoside-Rg1"[tiab]
  OR "ginsenoside Rb1"[tiab]
  OR "ginsenoside-Rb1"[tiab]
  OR "ginsenoside Rg2"[tiab]
  OR "ginsenoside Rg3"[tiab]
  OR "ginsenoside Rg5"[tiab]
  OR "ginsenoside Re"[tiab]
  OR "ginsenoside Rd"[tiab]
  OR "ginsenoside Rh2"[tiab]
  OR "compound K"[tiab]
  OR "ginsenoside compound K"[tiab]
  OR "notoginsenoside R1"[tiab]
  OR NGR1[tiab]
  OR "Panax ginseng extract*"[tiab]
  OR "ginseng berry"[tiab]
)
AND
(
  "Diabetes Mellitus"[Mesh]
  OR diabet*[tiab]
  OR hyperglycemi*[tiab]
  OR "high glucose"[tiab]
  OR "high-glucose"[tiab]
  OR "type 2 diabetes"[tiab]
  OR T2DM[tiab]
  OR "insulin resistance"[tiab]
  OR streptozotocin[tiab]
  OR STZ[tiab]
  OR "db/db"[tiab]
  OR "HFD/STZ"[tiab]
  OR alloxan[tiab]
  OR "advanced glycation"[tiab]
  OR AGEs[tiab]
  OR methylglyoxal[tiab]
)
AND
(
  "Cognition Disorders"[Mesh]
  OR "Memory Disorders"[Mesh]
  OR "diabetes-associated cognitive impairment"[tiab]
  OR "diabetes-associated cognitive dysfunction"[tiab]
  OR "diabetic cognitive impairment"[tiab]
  OR "diabetic cognitive dysfunction"[tiab]
  OR "diabetic encephalopathy"[tiab]
  OR "diabetic brain"[tiab]
  OR "diabetic Alzheimer's disease"[tiab]
  OR "cognitive impairment"[tiab]
  OR "cognitive dysfunction"[tiab]
  OR "memory impairment"[tiab]
  OR "learning and memory"[tiab]
  OR hippocamp*[tiab]
  OR "hippocampal neuron*"[tiab]
  OR neuron*[tiab]
  OR "brain insulin resistance"[tiab]
  OR neuroinflammation[tiab]
  OR "blood-brain barrier"[tiab]
  OR "blood brain barrier"[tiab]
  OR BBB[tiab]
)
```

Database: PubMed. Historical search date: 3 July 2026, 10:48. Historical hits: 151. Targeted update: 25 August 2026, 152 records; PMID 42577123 was the only additional record and was excluded at title/abstract screening. The screenshot below is the verified historical search result captured on 3 July 2026.

Figure S1.1. Screenshot of the PubMed search on 3 July 2026 showing 151 records (historical baseline).

Targeted PubMed update. The 25 August 2026 targeted update returned 152 records; PMID 42577123 was the only additional record and was excluded at title/abstract screening. The final 59-record evidence set is unchanged.

### S1.2. Embase: verified single search string

```
(
  'ginsenoside'/exp
  OR 'panax'/exp
  OR ginsenoside*:ti,ab,kw
  OR notoginsenoside*:ti,ab,kw
  OR panaxoside*:ti,ab,kw
  OR 'ginseng saponin*':ti,ab,kw
  OR 'panax saponin*':ti,ab,kw
  OR 'panax ginseng saponin*':ti,ab,kw
  OR 'panax notoginseng saponin*':ti,ab,kw
  OR 'notoginseng saponin*':ti,ab,kw
  OR 'total saponins of panax notoginseng':ti,ab,kw
  OR pns:ti,ab,kw
  OR 'ginsenoside rg1':ti,ab,kw
  OR 'ginsenoside rb1':ti,ab,kw
  OR 'ginsenoside rg2':ti,ab,kw
  OR 'ginsenoside rg3':ti,ab,kw
  OR 'ginsenoside rg5':ti,ab,kw
  OR 'ginsenoside re':ti,ab,kw
  OR 'ginsenoside rd':ti,ab,kw
  OR 'ginsenoside rh2':ti,ab,kw
  OR 'compound k':ti,ab,kw
  OR 'ginsenoside compound k':ti,ab,kw
  OR 'notoginsenoside r1':ti,ab,kw
  OR ngr1:ti,ab,kw
  OR 'panax ginseng extract*':ti,ab,kw
  OR 'ginseng berry':ti,ab,kw
)
AND
(
  'diabetes mellitus'/exp
  OR diabet*:ti,ab,kw
  OR hyperglycemi*:ti,ab,kw
  OR 'high glucose':ti,ab,kw
  OR 'high-glucose':ti,ab,kw
  OR 'type 2 diabetes':ti,ab,kw
  OR t2dm:ti,ab,kw
  OR 'insulin resistance':ti,ab,kw
  OR streptozotocin:ti,ab,kw
  OR stz:ti,ab,kw
  OR 'db/db':ti,ab,kw
  OR 'hfd/stz':ti,ab,kw
  OR alloxan:ti,ab,kw
  OR 'advanced glycation':ti,ab,kw
  OR ages:ti,ab,kw
  OR methylglyoxal:ti,ab,kw
)
AND
(
  'cognitive defect'/exp
  OR 'memory disorder'/exp
  OR 'diabetes-associated cognitive impairment':ti,ab,kw
  OR 'diabetes-associated cognitive dysfunction':ti,ab,kw
  OR 'diabetic cognitive impairment':ti,ab,kw
  OR 'diabetic cognitive dysfunction':ti,ab,kw
  OR 'diabetic encephalopathy':ti,ab,kw
  OR 'diabetic brain':ti,ab,kw
  OR 'diabetic alzheimer*':ti,ab,kw
  OR 'cognitive impairment':ti,ab,kw
  OR 'cognitive dysfunction':ti,ab,kw
  OR 'memory impairment':ti,ab,kw
  OR 'learning and memory':ti,ab,kw
  OR hippocamp*:ti,ab,kw
  OR 'hippocampal neuron*':ti,ab,kw
  OR neuron*:ti,ab,kw
  OR 'brain insulin resistance':ti,ab,kw
  OR neuroinflammation:ti,ab,kw
  OR 'blood-brain barrier':ti,ab,kw
  OR 'blood brain barrier':ti,ab,kw
  OR bbb:ti,ab,kw
)
```

Database: Embase. Historical search date: 3 July 2026, 10:56. Historical hits: 403. Status: Historical baseline final. The screenshot below is the verified historical search result captured on 3 July 2026.

Figure S1.2. Screenshot of the Embase search on 3 July 2026 showing 403 records (historical baseline).

### S1.3. Web of Science Core Collection: verified single search string

```
TS=(
  (
    ginsenoside*
    OR notoginsenoside*
    OR panaxoside*
    OR "ginseng saponin*"
    OR "Panax saponin*"
    OR "Panax ginseng saponin*"
    OR "Panax notoginseng saponin*"
    OR "notoginseng saponin*"
    OR "total saponins of Panax notoginseng"
    OR PNS
    OR "ginsenoside Rg1"
    OR "ginsenoside Rb1"
    OR "ginsenoside Rg2"
    OR "ginsenoside Rg3"
    OR "ginsenoside Rg5"
    OR "ginsenoside Re"
    OR "ginsenoside Rd"
    OR "ginsenoside Rh2"
    OR "compound K"
    OR "ginsenoside compound K"
    OR "notoginsenoside R1"
    OR NGR1
    OR "Panax ginseng extract*"
    OR "ginseng berry"
  )
  AND
  (
    diabet*
    OR hyperglycemi*
    OR "high glucose"
    OR "high-glucose"
    OR "type 2 diabetes"
    OR T2DM
    OR "insulin resistance"
    OR streptozotocin
    OR STZ
    OR "db/db"
    OR "HFD/STZ"
    OR alloxan
    OR "advanced glycation"
    OR AGEs
    OR methylglyoxal
  )
  AND
  (
    "diabetes-associated cognitive impairment"
    OR "diabetes-associated cognitive dysfunction"
    OR "diabetic cognitive impairment"
    OR "diabetic cognitive dysfunction"
    OR "diabetic encephalopathy"
    OR "diabetic brain"
    OR "diabetic Alzheimer*"
    OR "cognitive impairment"
    OR "cognitive dysfunction"
    OR "memory impairment"
    OR "learning and memory"
    OR hippocamp*
    OR "hippocampal neuron*"
    OR neuron*
    OR "brain insulin resistance"
    OR neuroinflammation
    OR "blood-brain barrier"
    OR "blood brain barrier"
    OR BBB
  )
)
```

Database: Web of Science Core Collection. Historical search date: 3 July 2026, 11:42. Historical hits: 518. Status: Historical baseline final. The screenshot below is the verified historical search result captured on 3 July 2026.

Figure S1.3. Screenshot of the Web of Science Core Collection search on 3 July 2026 showing 518 records (historical baseline).

### S1.4. Scopus: verified single search string

```
TITLE-ABS-KEY(
  (
    ginsenoside*
    OR notoginsenoside*
    OR panaxoside*
    OR "ginseng saponin*"
    OR "Panax saponin*"
    OR "Panax ginseng saponin*"
    OR "Panax notoginseng saponin*"
    OR "notoginseng saponin*"
    OR "total saponins of Panax notoginseng"
    OR PNS
    OR "ginsenoside Rg1"
    OR "ginsenoside Rb1"
    OR "ginsenoside Rg2"
    OR "ginsenoside Rg3"
    OR "ginsenoside Rg5"
    OR "ginsenoside Re"
    OR "ginsenoside Rd"
    OR "ginsenoside Rh2"
    OR "compound K"
    OR "ginsenoside compound K"
    OR "notoginsenoside R1"
    OR NGR1
    OR "Panax ginseng extract*"
    OR "ginseng berry"
  )
  AND
  (
    diabet*
    OR hyperglycemi*
    OR "high glucose"
    OR "high-glucose"
    OR "type 2 diabetes"
    OR T2DM
    OR "insulin resistance"
    OR streptozotocin
    OR STZ
    OR "db/db"
    OR "HFD/STZ"
    OR alloxan
    OR "advanced glycation"
    OR AGEs
    OR methylglyoxal
  )
  AND
  (
    "diabetes-associated cognitive impairment"
    OR "diabetes-associated cognitive dysfunction"
    OR "diabetic cognitive impairment"
    OR "diabetic cognitive dysfunction"
    OR "diabetic encephalopathy"
    OR "diabetic brain"
    OR "diabetic Alzheimer*"
    OR "cognitive impairment"
    OR "cognitive dysfunction"
    OR "memory impairment"
    OR "learning and memory"
    OR hippocamp*
    OR "hippocampal neuron*"
    OR neuron*
    OR "brain insulin resistance"
    OR neuroinflammation
    OR "blood-brain barrier"
    OR "blood brain barrier"
    OR BBB
  )
)
```

Database: Scopus. Historical search date: 3 July 2026, 11:48. Historical hits: 524. Status: Historical baseline final. The screenshot below is the verified historical search result captured on 3 July 2026.

Figure S1.4. Screenshot of the Scopus search on 3 July 2026 showing 524 records (historical baseline).

### S1.5. CNKI: verified single search string

SU=('人参皂苷'+'人参皂甙'+'三七皂苷'+'三七总皂苷'+'人参总皂苷'+'原人参二醇'+'原人参三醇'+'化合物K'+'Compound K'+'人参皂苷Rg1'+'人参皂苷Rb1'+'人参皂苷Rg2'+'人参皂苷Rg3'+'人参皂苷Rg5'+'人参皂苷Re'+'人参皂苷Rd'+'人参皂苷Rh2'+'三七皂苷R1'+'Notoginsenoside R1'+'NGR1')
*
('糖尿病'+'2型糖尿病'+'高糖'+'高血糖'+'胰岛素抵抗'+'链脲佐菌素'+'STZ'+'db/db'+'糖基化终末产物'+'甲基乙二醛')
*
('糖尿病认知障碍'+'糖尿病相关认知障碍'+'糖尿病性认知障碍'+'糖尿病认知功能障碍'+'糖尿病脑病'+'糖尿病脑损伤'+'认知障碍'+'认知功能障碍'+'记忆障碍'+'学习记忆'+'海马'+'海马神经元'+'神经元损伤'+'脑胰岛素抵抗'+'神经炎症'+'血脑屏障'+'阿尔茨海默')

Database: CNKI. Historical search date: 3 July 2026, 12:00. Historical hits: 20. Status: Historical baseline final. The screenshot below is the verified historical search result captured on 3 July 2026.

Figure S1.5. Screenshot of the CNKI search on 3 July 2026 showing 20 records (historical baseline).

### S1.6. Wanfang: verified single search string

主题:(("人参皂苷" OR "人参皂甙" OR "三七皂苷" OR "三七总皂苷" OR "人参总皂苷" OR "原人参二醇" OR "原人参三醇" OR "化合物K" OR "Compound K" OR "人参皂苷Rg1" OR "人参皂苷Rb1" OR "人参皂苷Rg2" OR "人参皂苷Rg3" OR "人参皂苷Rg5" OR "人参皂苷Re" OR "人参皂苷Rd" OR "人参皂苷Rh2" OR "三七皂苷R1" OR "Notoginsenoside R1" OR "NGR1")
AND
("糖尿病" OR "2型糖尿病" OR "高糖" OR "高血糖" OR "胰岛素抵抗" OR "链脲佐菌素" OR "STZ" OR "db/db" OR "糖基化终末产物" OR "甲基乙二醛")
AND
("糖尿病认知障碍" OR "糖尿病相关认知障碍" OR "糖尿病性认知障碍" OR "糖尿病认知功能障碍" OR "糖尿病脑病" OR "糖尿病脑损伤" OR "认知障碍" OR "认知功能障碍" OR "记忆障碍" OR "学习记忆" OR "海马" OR "海马神经元" OR "神经元损伤" OR "脑胰岛素抵抗" OR "神经炎症" OR "血脑屏障" OR "阿尔茨海默"))

Database: Wanfang. Historical search date: 3 July 2026, 12:02. Historical hits: 40. Status: Historical baseline final. The screenshot below is the verified historical search result captured on 3 July 2026.

Figure S1.6. Screenshot of the Wanfang search on 3 July 2026 showing 40 records (historical baseline).

### S1.7. VIP: verified single search string

M=(人参皂苷+人参皂甙+三七皂苷+三七总皂苷+人参总皂苷+原人参二醇+原人参三醇+化合物K+Compound K+人参皂苷Rg1+人参皂苷Rb1+人参皂苷Rg2+人参皂苷Rg3+人参皂苷Rg5+人参皂苷Re+人参皂苷Rd+人参皂苷Rh2+三七皂苷R1+Notoginsenoside R1+NGR1)
*
(糖尿病+2型糖尿病+高糖+高血糖+胰岛素抵抗+链脲佐菌素+STZ+db/db+糖基化终末产物+甲基乙二醛)
*
(糖尿病认知障碍+糖尿病相关认知障碍+糖尿病性认知障碍+糖尿病认知功能障碍+糖尿病脑病+糖尿病脑损伤+认知障碍+认知功能障碍+记忆障碍+学习记忆+海马+海马神经元+神经元损伤+脑胰岛素抵抗+神经炎症+血脑屏障+阿尔茨海默)

Database: VIP. Historical search date: 3 July 2026, 12:03. Historical hits: 43. Status: Historical baseline final. The screenshot below is the verified historical search result captured on 3 July 2026.

Figure S1.7. Screenshot of the VIP search on 3 July 2026 showing 43 records (historical baseline).

### S1.8. SinoMed: verified single search string

("人参皂苷" OR "人参皂甙" OR "三七皂苷" OR "三七总皂苷" OR "人参总皂苷" OR "原人参二醇" OR "原人参三醇" OR "化合物K" OR "Compound K" OR "人参皂苷Rg1" OR "人参皂苷Rb1" OR "人参皂苷Rg2" OR "人参皂苷Rg3" OR "人参皂苷Rg5" OR "人参皂苷Re" OR "人参皂苷Rd" OR "人参皂苷Rh2" OR "三七皂苷R1" OR "Notoginsenoside R1" OR "NGR1")
AND
("糖尿病" OR "2型糖尿病" OR "高糖" OR "高血糖" OR "胰岛素抵抗" OR "链脲佐菌素" OR "STZ" OR "db/db" OR "糖基化终末产物" OR "甲基乙二醛")
AND
("糖尿病认知障碍" OR "糖尿病相关认知障碍" OR "糖尿病性认知障碍" OR "糖尿病认知功能障碍" OR "糖尿病脑病" OR "糖尿病脑损伤" OR "认知障碍" OR "认知功能障碍" OR "记忆障碍" OR "学习记忆" OR "海马" OR "海马神经元" OR "神经元损伤" OR "脑胰岛素抵抗" OR "神经炎症" OR "血脑屏障" OR "阿尔茨海默")

Database: SinoMed. Historical search date: 3 July 2026, 12:06. Historical hits: 11. Status: Historical baseline final. The screenshot below is the verified historical search result captured on 3 July 2026.

Figure S1.8. Screenshot of the SinoMed search on 3 July 2026 showing 11 records (historical baseline).

### S1.9. Cochrane CENTRAL (supplementary search): verified single search string

```
(
  ginsenoside*
  OR notoginsenoside*
  OR panaxoside*
  OR "Panax notoginseng saponins"
  OR PNS
  OR "ginsenoside Rg1"
  OR "ginsenoside Rb1"
  OR "ginsenoside Rg2"
  OR "ginsenoside Rg3"
  OR "ginsenoside Re"
  OR "ginsenoside Rd"
  OR "compound K"
  OR "notoginsenoside R1"
  OR "Panax ginseng extract"
):ti,ab,kw
AND
(
  diabet*
  OR "high glucose"
  OR "type 2 diabetes"
  OR "insulin resistance"
  OR streptozotocin
  OR STZ
  OR "db/db"
):ti,ab,kw
AND
(
  "diabetes-associated cognitive impairment"
  OR "diabetes-associated cognitive dysfunction"
  OR "diabetic cognitive impairment"
  OR "diabetic cognitive dysfunction"
  OR "diabetic encephalopathy"
  OR "cognitive impairment"
  OR "cognitive dysfunction"
  OR "memory impairment"
  OR hippocamp*
  OR "blood-brain barrier"
):ti,ab,kw
```

Database: Cochrane CENTRAL (supplementary source). Historical search date: 3 July 2026, 11:57. Historical hits: 2. Status: Historical status confirmed. The screenshot below is the verified historical search result captured on 3 July 2026.

Figure S1.9. Screenshot of the Cochrane CENTRAL supplementary search on 3 July 2026 showing 2 records (historical baseline).

## Supplementary Table S2. Evidence inventory of the 59 full-text records

| **Reference** | **Title** | **Evidence tier** | **Permitted use** |
| --- | --- | --- | --- |
| Fu et al., 2026 | TRPC6-TXNIP-NLRP3 signaling axis is involved in type 2 diabetes-associated cognitive dysfunction and the intervention of ginsenoside Rg1 | A1: direct or near-direct in vivo evidence relevant to DACD | Supports behavioral claims when central endpoints are present |
| Li et al., 2025 | Notoginsenoside R1, a Novel Natural PPARγ Agonist, Attenuates Cognitive Deficits in a Mouse Model of Diabetic Alzheimer's Disease Through Enhancing GLUT4-Dependent Neuronal Glucose Uptake | A1: direct or near-direct in vivo evidence relevant to DACD | Supports behavioral claims when central endpoints are present |
| Su et al., 2024 | Molecular mechanism of ginsenoside Rg1 alleviating cognitive impairment in T2DM rats | A1: direct or near-direct in vivo evidence relevant to DACD | Supports behavioral claims when central endpoints are present |
| Dong et al., 2023 | Ginsenoside Rg1 treatment protects against cognitive dysfunction via inhibiting PLC-CN-NFAT1 signaling in T2DM mice | A1: direct or near-direct in vivo evidence relevant to DACD | Supports behavioral claims when central endpoints are present |
| Huang et al., 2023 | Ginsenoside Rg1 attenuates type 2 diabetes-induced neuronal damage by inhibiting MAPK-NLRP1 pathway | A1: direct or near-direct in vivo evidence relevant to DACD | Supports behavioral claims when central endpoints are present |
| Li et al., 2020 | Effects of compound K, a metabolite of ginsenosides, on memory and cognitive dysfunction in db/db mice involve the inhibition of ER stress and the NLRP3 inflammasome pathway | A1: direct or near-direct in vivo evidence relevant to DACD | Supports behavioral claims when central endpoints are present |
| Yang et al., 2020 | Ginsenoside Rb1 Improves Cognitive Impairment Induced by Insulin Resistance through Cdk5/p35-NMDAR-IDE Pathway | A1: direct or near-direct in vivo evidence relevant to DACD | Supports behavioral claims when central endpoints are present |
| Tian et al., 2018 | Ginsenoside Ameliorates Cognitive Dysfunction in Type 2 Diabetic Goto-Kakizaki Rats | A1: direct or near-direct in vivo evidence relevant to DACD | Supports behavioral claims when central endpoints are present |
| Zhai et al., 2018 | Notoginsenoside R1 ameliorates diabetic encephalopathy by activating the Nrf2 pathway and inhibiting NLRP3 inflammasome activation | A1 (in vivo); A2 (cell) | A1: supports behavioral claims when central endpoints are present; A2: mechanistic support only |
| T. Zheng et al., 2018 | Effect and possible mechanism of panaxtrial saponins on cognitive dysfunction in diabetic rats | A1: direct or near-direct in vivo evidence relevant to DACD | Supports behavioral claims when central endpoints are present |
| Kim et al., 2017 | Ginsenoside Re Ameliorates Brain Insulin Resistance and Cognitive Dysfunction in High Fat Diet-Induced C57BL/6 Mice | A1: direct or near-direct in vivo evidence relevant to DACD | Supports behavioral claims when central endpoints are present |
| Li et al., 2015 | Roles of panaxtrial saponins in cognition and memory of diabetic rat and in activity of astrocytes | A1: direct or near-direct in vivo evidence relevant to DACD | Supports behavioral claims when central endpoints are present |
| Liao et al., 2014 | Research on protective function of Panax Notoginseng saponins for spatial cognitive functions and hippocampal neurons in diabetic mice | A1: direct or near-direct in vivo evidence relevant to DACD | Supports behavioral claims when central endpoints are present |
| Gao et al., 2014 | Effect and Mechanism of Ginsenosides Rb1 on Insulin Signaling Pathway in Type 2 Diabetes Mice with Memory Impairment | A1: direct or near-direct in vivo evidence relevant to DACD | Supports behavioral claims when central endpoints are present |
| Mao et al., 2014 | Effect and Mechanism of Ginsenosides Rb1 and Rosiglitazone on Ethology and Insulin Signaling Pathway of Type 2 Diabetes Mice with Cognitive Impairment | A1: direct or near-direct in vivo evidence relevant to DACD | Supports behavioral claims when central endpoints are present |
| Liu et al., 2012 | Ginsenoside Re attenuates diabetes-associated cognitive deficits in rats | A1: direct or near-direct in vivo evidence relevant to DACD | Supports behavioral claims when central endpoints are present |
| Zhu et al., 2026 | Ginsenoside Rg2 Targeting PDHE1α to Improve High Glucose-Induced Hippocampal Neuronal Damage Based on CETSA Experiment | A2 diabetes-like neural-cell mechanism | Mechanistic support only; no behavioral efficacy |
| Nan et al., 2019 | Ginsenoside Rb1 mitigates oxidative stress and apoptosis induced by methylglyoxal in SH-SY5Y cells via the PI3K/Akt pathway | A2 diabetes-like neural-cell mechanism | Mechanistic support only; no behavioral efficacy |
| Gu et al., 2014 | [Effect of ginsenoside Rb1 on insulin signal transduction pathway in hippocampal neurons of high-glucose-fed rats] | A2 diabetes-like neural-cell mechanism | Mechanistic support only; no behavioral efficacy |
| Liu et al., 2014 | Ginsenoside Rb1 protects hippocampal neurons from high glucose-induced neurotoxicity by inhibiting GSK3β-mediated CHOP induction | A2 diabetes-like neural-cell mechanism | Mechanistic support only; no behavioral efficacy |
| J.M. Chu et al., 2014 | Ginsenosides attenuate methylglyoxal-induced impairment of insulin signaling and subsequent apoptosis in primary astrocytes | A2 diabetes-like neural-cell mechanism | Mechanistic support only; no behavioral efficacy |
| Liu et al., 2013 | Neuroprotective effects of ginsenoside Rb1 on high glucose-induced neurotoxicity in primary cultured rat hippocampal neurons | A2 diabetes-like neural-cell mechanism | Mechanistic support only; no behavioral efficacy |
| Chen et al., 2026b | Tianhuang Formula and its active ingredient GsRg1 ameliorate central insulin resistance and oxidative stress by enhancing hypothalamic autophagy | B auxiliary Panax/BBB/formulation | Contextual support; no transfer to purified-monomer efficacy |
| She et al., 2026 | Wumei Pill ameliorates cognitive dysfunction in diabetic encephalopathy via inhibiting astrocyte-derived chemokine-receptor mediated neuroinflammation | B auxiliary Panax/BBB/formulation | Contextual support; no transfer to purified-monomer efficacy |
| Cui et al., 2024 | Study on the Mechanism of Panax Quinquefolium-Acorus Calamus Ameliorating Diabetic EncepHalopathy in Mice by Mediating Nrf2-Keap1 Signaling Pathway | B auxiliary Panax/BBB/formulation | Contextual support; no transfer to purified-monomer efficacy |
| Huan et al., 2023 | Hippocampal proteins discovery of Panax quinquefolius and Acorus gramineus ameliorating cognitive impairment in diabetic rats | B auxiliary Panax/BBB/formulation | Contextual support; no transfer to purified-monomer efficacy |
| Zhang et al., 2021 | Berberine and Ginsenoside Rb1 Ameliorate Depression-Like Behavior in Diabetic Rats | B auxiliary Panax/BBB/formulation | Contextual support; no transfer to purified-monomer efficacy |
| Ye et al., 2021 | Huang-Pu-Tong-Qiao Formula Ameliorates the Hippocampus Apoptosis in Diabetic Cognitive Dysfunction Mice by Activating CREB/BDNF/TrkB Signaling Pathway | B auxiliary Panax/BBB/formulation | Contextual support; no transfer to purified-monomer efficacy |
| Yang et al., 2021 | Metabolomics study of cerebrospinal fluid from diabetic rats with cognitive impairment simultaneously treated with Panax quinquefolius and Acorus gramineus | B auxiliary Panax/BBB/formulation | Contextual support; no transfer to purified-monomer efficacy |
| Lee et al., 2020 | Ginseng Extracts, GS-KG9 and GS-E3D, Prevent Blood-Brain Barrier Disruption and Thereby Inhibit Apoptotic Cell Death of Hippocampal Neurons in Streptozotocin-Induced Diabetic Rats | B auxiliary Panax/BBB/formulation | Contextual support; no transfer to purified-monomer efficacy |
| Ahmed et al., 2020 | Jiawei Shengmai San herbal formula ameliorates diabetic associate cognitive decline by modulating AKT and CREB in rats | B auxiliary Panax/BBB/formulation | Contextual support; no transfer to purified-monomer efficacy |
| Chu et al., 2018 | HMGB1 is involved into the anti-ischemic stroke effect of ginsenoside RG1 in diabetic rats | B auxiliary Panax/BBB/formulation | Contextual support; no transfer to purified-monomer efficacy |
| Shen et al., 2017 | Ginsenoside Rg1 nanoparticle penetrating the blood-brain barrier to improve the cerebral function of diabetic rats complicated with cerebral infarction | B auxiliary Panax/BBB/formulation | Contextual support; no transfer to purified-monomer efficacy |
| Park et al., 2015 | Effect of Ginseng (Panax ginseng) Berry EtOAc Fraction on Cognitive Impairment in C57BL/6 Mice under High-Fat Diet Inducement | B auxiliary Panax/BBB/formulation | Contextual support; no transfer to purified-monomer efficacy |
| Tan et al., 2015 | Ginseng improves cognitive deficit via the RAGE/NF-κB pathway in advanced glycation end product-induced rats | B auxiliary Panax/BBB/formulation | Contextual support; no transfer to purified-monomer efficacy |
| S. Chu et al., 2014 | Ginsenoside Rg5 improves cognitive dysfunction and beta-amyloid deposition in STZ-induced memory impaired rats via attenuating neuroinflammatory responses | B auxiliary Panax/BBB/formulation | Contextual support; no transfer to purified-monomer efficacy |
| Chang et al., 2004 | Administration of Ginseng radix decreases nitric oxide synthase expression in the hippocampus of streptozotocin-induced diabetic rats | B auxiliary Panax/BBB/formulation | Contextual support; no transfer to purified-monomer efficacy |
| Jang et al., 2003 | Effect of ginseng radix on c-Fos expression in the hippocampus of streptozotocin-induced diabetic rats | B auxiliary Panax/BBB/formulation | Contextual support; no transfer to purified-monomer efficacy |
| Lim et al., 2002 | Ginseng radix increases cell proliferation in dentate gyrus of rats with streptozotocin-induced diabetes | B auxiliary Panax/BBB/formulation | Contextual support; no transfer to purified-monomer efficacy |
| Chen et al., 2025 | Mechanisms underlying cognitive impairment and management strategies in type 2 diabetes | C contextual pharmacology/mechanism | Disease/pharmacology context only |
| Sun et al., 2026 | Ginsenosides in Modern Pharmaceutics: Mechanisms, Applications, Challenges, and Perspectives | C contextual pharmacology/mechanism | Disease/pharmacology context only |
| Wen et al., 2025 | Pharmacokinetics and Biological Activities of Notoginsenoside R1: A Systematical Review | C contextual pharmacology/mechanism | Disease/pharmacology context only |
| Wu et al., 2024 | Ginsenoside Rg1, lights up the way for the potential prevention of Alzheimer's disease due to its therapeutic effects on the drug-controllable risk factors of Alzheimer's disease | C contextual pharmacology/mechanism | Disease/pharmacology context only |
| Choi and Kim, 2023 | Compound K - An immunomodulator of macrophages in inflammation | C contextual pharmacology/mechanism | Disease/pharmacology context only |
| Liu et al., 2023 | Inhibition of the pyroptosis-associated inflammasome pathway: The important potential mechanism of ginsenosides in ameliorating diabetes and its complications | C contextual pharmacology/mechanism | Disease/pharmacology context only |
| Gao et al., 2022 | Pharmacological Properties of Ginsenoside Re | C contextual pharmacology/mechanism | Disease/pharmacology context only |
| Lin et al., 2022 | Recent progress (2015-2020) in the investigation of the pharmacological effects and mechanisms of ginsenoside Rb(1), a main active ingredient in Panax ginseng Meyer | C contextual pharmacology/mechanism | Disease/pharmacology context only |
| Wang et al., 2022 | Recent Studies on the Pharmacological Activities and Structural Modifications of Compound-K | C contextual pharmacology/mechanism | Disease/pharmacology context only |
| Ghafouri-Fard et al., 2022 | The effects of Ginsenosides on PI3K/AKT signaling pathway | C contextual pharmacology/mechanism | Disease/pharmacology context only |
| Li et al., 2021 | Neuroprotective Potentials of Panax Ginseng Against Alzheimer's Disease: A Review of Preclinical and Clinical Evidences | C contextual pharmacology/mechanism | Disease/pharmacology context only |
| Liu et al., 2020 | Focus on Notoginsenoside R1 in Metabolism and Prevention Against Human Diseases | C contextual pharmacology/mechanism | Disease/pharmacology context only |
| Qu et al., 2020 | Panax notoginseng saponins and their applications in nervous system disorders: a narrative review | C contextual pharmacology/mechanism | Disease/pharmacology context only |
| Szczuka et al., 2019 | American Ginseng (Panax quinquefolium L.) as a Source of Bioactive Phytochemicals with Pro-Health Properties | C contextual pharmacology/mechanism | Disease/pharmacology context only |
| Zhou et al., 2019 | Ginsenoside Rb1 as an Anti-Diabetic Agent and Its Underlying Mechanism Analysis | C contextual pharmacology/mechanism | Disease/pharmacology context only |
| Jakaria et al., 2018 | Active ginseng components in cognitive impairment: Therapeutic potential and prospects for delivery and clinical study | C contextual pharmacology/mechanism | Disease/pharmacology context only |
| Mohanan et al., 2018 | Molecular signaling of ginsenosides Rb1, Rg1, and Rg3 and their mode of actions | C contextual pharmacology/mechanism | Disease/pharmacology context only |
| Bai et al., 2018 | Therapeutic Potential of Ginsenosides as an Adjuvant Treatment for Diabetes | C contextual pharmacology/mechanism | Disease/pharmacology context only |
| Mancuso and Santangelo, 2017 | Panax ginseng and Panax quinquefolius: From pharmacology to toxicology | C contextual pharmacology/mechanism | Disease/pharmacology context only |
| Geng et al., 2010 | Ginseng for cognition | C contextual pharmacology/mechanism | Disease/pharmacology context only |

Note: Each retained report is listed once in this inventory. Zhai et al. 2018 contains both A1 in vivo and A2 neural-cell components; it is listed as A1 (in vivo); A2 (cell), counted once in the 16-report A1 inventory, and appraised in Supplementary Table S3.

## Supplementary Table S3. Study-level appraisal of A1 and A2 evidence

NR, not reported. Appraisal was evidence-type specific and organized across four domains: design/reporting, material definition, causal validation, and principal limitation. The domains calibrated claim strength and were not combined into an unvalidated numerical score. Absence of reporting was not interpreted as proof that a procedure was not performed. Zhai et al. 2018 is appraised once as a single report containing A1 in vivo and A2 neural-cell components.

| **Study** | **Tier** | **Intervention** | **Model/system** | **Design/reporting** | **Material definition** | **Causal validation** | **Principal limitation** | Dose, route, and duration |
| --- | --- | --- | --- | --- | --- | --- | --- | --- |
| Fu et al., 2026 | A1 | Rg1 | HFD/STZ T2DM mice; *Trpc6*−/− | Random allocation and blinded assessment reported; n = 12/group | CAS and HPLC purity ≥98% | *Trpc6* knockout | No brain exposure or direct Rg1-TRPC6 binding | 10 mg/kg/day, oral gavage, 8 weeks |
| Li et al., 2025 | A1 | NGR1 | APP/PS1×db/db mice; primary neurons | Randomized, double-blind design; dose-response and mechanistic cohorts | Purified NGR1; formulation reported | SPR, reporter assay, GW9662 antagonism | Diabetic AD-like model limits generalization to DACD | 10–80 mg/kg/day; mechanistic dose 40 mg/kg/day, intragastric, 16 weeks |
| Su et al., 2024 | A1 | Rg1 | HFD/STZ T2DM rats | Dose-response design; randomization/blinding not clearly reported | Compound source reported; full batch characterization limited | No pathway perturbation | Central action not separated from systemic metabolic benefit | 25, 50, or 100 mg/kg/day, intragastric, 8 weeks |
| Dong et al., 2023 | A1 | Rg1 | HFD/STZ T2DM mice | Random allocation; multiple behavioral and tissue readouts; blinding unclear | Purity >98% | No inhibitor, knockdown, or binding assay | Pathway evidence mainly associative | 1, 5, or 10 mg/kg, oral, 8 weeks |
| Huang et al., 2023 | A1 | Rg1 | HFD/STZ T2DM mice | Random allocation reported; blinding and sample-size justification unclear | Source reported; purity/batch detail limited | Neuron/NLRP1 colocalization without pathway perturbation | Association does not establish MAPK-NLRP1 dependence | 1, 5, or 10 mg/kg, gavage, 8 weeks |
| Li et al., 2020 | A1 | CK | db/db mice | Randomized groups and double-blind behavioral testing | CK purity ≥98% | No direct pathway perturbation | No brain exposure; ER stress/NLRP3 changes remain associative | 10 mg/kg/day, oral, 12 weeks |
| Yang et al., 2020 | A1 | Rb1 | STZ-associated cognitive model | Random allocation reported; two behavioral tasks; blinding unclear | Commercial Rb1 source reported; batch details limited | No target rescue | High-dose STZ model and no direct engagement | 30 mg/kg/day, oral; MWM at week 4 |
| Tian et al., 2018 | A1 | Total ginsenosides | Goto-Kakizaki rats | Random allocation; behavior plus brain biochemistry; blinding unclear | Total-ginsenoside composition insufficiently resolved | No causal perturbation | Fraction-level results cannot be assigned to a monomer | 20 mg/kg/day, intragastric, 10 weeks |
| Zhai et al., 2018 | A1/A2 (same report; in vivo and cell components) | NGR1 | db/db mice; high-glucose HT22 cells | In vivo and cell convergence; randomization/blinding incompletely reported | Defined NGR1 purity | LY294002 in cells | Causal test was cell based; exposure not measured | 10 or 30 mg/kg/day, oral, 10 weeks |
| T. Zheng et al., 2018 | A1 | PTS | STZ diabetic rats | Short report; behavior and histology; allocation/blinding unclear | PTS fraction not chemically profiled | No causal perturbation | Dose reported per rat rather than per body weight | 200 mg per rat/day, gavage, 3 months (as reported) |
| Kim et al., 2017 | A1 | Re | HFD-fed mice | Dose-response design; behavior and brain signaling; blinding unclear | Re purity >90% | No causal perturbation | Short metabolic model; peripheral contribution unresolved | 5, 10, or 20 mg/kg/day, intragastric, 4 weeks |
| Li et al., 2015 | A1 | PTS | STZ diabetic rats | n = 8/group; behavior and glial readouts; blinding unclear | PTS fraction not chemically profiled | No causal perturbation | Dose not weight normalized and material definition limited | 100 mg per rat twice daily, gavage, 3 months (as reported) |
| Liao et al., 2014 | A1 | PNS | STZ diabetic mice | Random allocation; behavior plus histology; blinding unclear | PNS fraction insufficiently characterized | No causal perturbation | Older fraction study lacks current chemical reporting | 200 mg/kg/day, gavage, 30 days |
| Gao et al., 2014 | A1 | Rb1 + rosiglitazone | KKay mice | Model animals stratified by glycemia; dose groups; blinding unclear | Rb1 purity reported; combination regimen defined | No monotherapy arm for Rb1 | Cannot attribute outcome to Rb1 alone | Rb1 15, 30, or 60 mg/kg + rosiglitazone 3 mg/kg, gavage, 6 weeks |
| Mao et al., 2014 | A1 | Rb1 + rosiglitazone | KKay mice | Model animals randomized after glycemic assessment; blinding unclear | Rb1 purity reported; combination regimen defined | No monotherapy arm for Rb1 | Cannot attribute outcome to Rb1 alone | Rb1 15, 30, or 60 mg/kg + rosiglitazone 3 mg/kg, gavage, 42 days |
| Liu et al., 2012 | A1 | Re | STZ diabetic rats | Behavior plus two brain regions; random allocation reported; blinding unclear | Re purity >90% | No causal perturbation | Systemic glucose lowering may contribute to cognition | 40 mg/kg, chronic treatment, 8 weeks |
| Zhu et al., 2026 | A2 | Rg2 | High-glucose HT22 cells | Dose/time-response and metabolic phenotyping | Defined G-Rg2; concentration series reported | SPR, CETSA, PDHE1α silencing | No animal behavior or *in vivo* brain exposure | 12.5, 25, or 50 μM; 24–72 h |
| Nan et al., 2019 | A2 | Rb1 | MGO-exposed SH-SY5Y cells | Dose-response and multiple apoptosis/redox assays; biological replication reported | Defined Rb1 source and concentration | LY294002 inhibition | Transformed cell line | 0.375–1.5 μM; 8-h pretreatment then 24-h MGO |
| Gu et al., 2014 | A2 | Rb1 | High-glucose primary hippocampal neurons | Primary culture and 72-h exposure; replication detail limited | Rb1 concentration reported | LiCl comparator | No direct target engagement; cell-only evidence | 1 μM, 72 h |
| Liu et al., 2014 | A2 | Rb1 | High-glucose primary hippocampal neurons | Primary culture and multiple ER-stress readouts | Rb1 concentration reported | LiCl pathway comparator | Cell-only evidence | 1 μM, 72 h |
| J.M. Chu et al., 2014 | A2 | Rd and R-Rh2 | MGO-exposed primary astrocytes | Primary culture, concentration range, and biological replication | Approximate purity 98%; HPLC/TLC verification | Dose-response without target perturbation | Astrocyte-only and subtype-specific evidence | 5–50 μM; 20 μM active range, 24 h |
| Liu et al., 2013 | A2 | Rb1 | High-glucose primary hippocampal neurons | Primary culture with viability, apoptosis, ER, and mitochondrial assays | Rb1 concentration reported | Mechanistic convergence without binding assay | Cell-only evidence | 1 μM, 72 h |

## Supplementary Table S4. SYRCLE-informed risk-of-bias assessment of the 16 A1 animal studies

| Study | Seq | Base | Conceal | Housing | Personnel blind | Random outcome | Assessor blind | Incomplete data | Selective reporting | Other bias |
| --- | --- | --- | --- | --- | --- | --- | --- | --- | --- | --- |
| Fu et al., 2026 | U | U | U | U | U | U | L | U | U | U |
| Li et al., 2025 | U | U | U | U | L | U | L | U | U | U |
| Su et al., 2024 | U | U | U | U | U | U | U | U | U | U |
| Dong et al., 2023 | U | U | U | U | U | U | U | U | U | U |
| Huang et al., 2023 | U | U | U | U | U | U | U | U | U | U |
| Li et al., 2020 | U | U | U | U | U | U | L | U | U | U |
| Yang et al., 2020 | U | U | U | U | U | U | U | U | U | U |
| Tian et al., 2018 | U | U | U | U | U | U | U | U | U | U |
| Zhai et al., 2018 | U | U | U | U | U | U | U | U | U | U |
| T. Zheng et al., 2018 | U | U | U | U | U | U | U | U | U | U |
| Kim et al., 2017 | U | U | U | U | U | U | U | U | U | U |
| Li et al., 2015 | L | U | U | U | U | U | U | U | U | U |
| Liao et al., 2014 | U | U | U | U | U | U | U | U | U | U |
| Gao et al., 2014 | U | U | U | U | U | U | U | U | U | H |
| Mao et al., 2014 | U | U | U | U | U | U | U | U | U | H |
| Liu et al., 2012 | U | U | U | U | U | U | U | U | U | U |

L, low risk; H, high risk; U, unclear risk. Seq, sequence generation; Base, baseline comparability; Conceal, allocation concealment. Unclear indicates that the publication did not provide enough information for a low- or high-risk judgment. No total score was calculated. The 'other bias' high-risk judgments for Gao et al. and Mao et al. reflect monomer-attribution confounding because every Rb1 group also received rosiglitazone. Sex, age, treatment timing, behavioral controls, material definition, and study-specific limitations are reported in main Table 2 and Supplementary Table S3.

| Translational modifier | What was recorded | Where recorded | Use in synthesis |
| --- | --- | --- | --- |
| Sex | Sex of animals was recorded per study; most A1 studies used male animals and one used females. | Table 2, column "Model/system, sex, and age" | Sex-dependent effects were rarely testable; limitation stated in Section 10.2 and Table 4. |
| Age and disease stage | Age at treatment and model maturity were recorded where reported. | Table 2, column "Model/system, sex, and age" | Young/adult preventive designs predominate; aged, multimorbid reversal remains untested. |
| Disease duration | Duration after diabetes induction or model phenotype was recorded where reported. | Table 2, columns "Treatment timing" and "Model/system, sex, and age" | Prevention/early treatment cannot be equated with reversal of established DACD. |
| Sample-size justification | Whether group size justification was reported was recorded. | Table S3 and Table S4 (where unclear) | Most A1 reports did not state a formal justification; interpreted as unclear, not as proof of absence. |
| Behavioral controls | Swimming-speed, open-field/locomotor, and sensory controls were recorded where reported. | Table 2, column "Cognitive test and central phenotype" | Controls were used to reduce motor or sensory confounds; they were not uniformly reported. |
| Treatment timing | Prevention, early treatment, or delayed treatment was recorded per study. | Table 2, column "Treatment timing" | Supports the conclusion that delayed-reversal designs are still needed. |
| Brain or CSF exposure | Quantitative central exposure was recorded where measured. | Table 4, column "Brain/CSF measurement in DACD" | Most A1 studies did not measure achieved brain exposure; central action remains unresolved. |
| Selective reporting | Domain-level risk was recorded as low, high, or unclear. | Table S4, column "Selective reporting" | Mostly unclear; no aggregate score was calculated. |

The following reporting-level summary is derived from the evidence inventory, study appraisal, risk-of-bias assessment, and exposure tables in this supplement. It records where each translational modifier was captured and how the modifier was used; it does not add new study-level claims beyond the values already reported in Tables 2, 4, S2, S3, and S4.

## Supplementary Table S5. Translational modifiers recorded for the A1 evidence
